# Supplementary material for: Missing data in amortized simulation-based neural posterior estimation
Source: PLoS Comput Biol. 2024 Jun 17;20(6):e1012184. doi: 10.1371/journal.pcbi.1012184 (PMC11213359; doi:10.1371/journal.pcbi.1012184)
Supplement: S1 Supplementary Information — (PDF) [file pcbi.1012184.s001.pdf]

S1 Supplementary Information: Missing data in amortized simulation-based neural posterior estimation

Zijian Wang[1], Jan Hasenauer[1,2,*], Yannik Schälte[1,2,3,*],

May 28, 2024

[1] University of Bonn, Life and Medical Sciences Institute, 53115 Bonn, Germany
[2] Helmholtz Center Munich, Computational Health Center, 85764 Neuherberg, Germany
[3] Technical University Munich, Center for Mathematics, 85748 Garching, Germany
[*] To whom correspondence should be addressed (jan.hasenauer@uni-bonn.de, yannik.schaelte@uni-bonn.de)

Contents

1 Simple deletion of missing data does not work **2**

2 Linear imputation of missing data does not work **3**

3 Can imputation give more informative posteriors? **4**

4 Model details **5**
4.1 Conversion reaction model . . . 5
4.2 Sinusoidal model . . . 6
4.3 FitzHugh-Nagumo model . . . 6
4.4 SIR ODE model . . . 7
4.5 SIR SSA model . . . 7
4.6 Conversion reaction model with parameter-dependent missingness . . . 8

5 Supplementary error analysis **9**
5.1 Methods . . . 9
5.2 Conversion reaction model . . . 10
5.3 Sinusoidal model . . . 13
5.4 FitzHugh-Nagumo model . . . 18
5.5 SIR ODE model . . . 30
5.6 SIR SSA model . . . 32
5.7 Conversion reaction model with parameter-dependent missingness . . . 35

1 Simple deletion of missing data does not work

The existing BayesFlow method allows to handle time series data of different length by converting them into a fixed-size vector of summary statistics using e.g. LSTM networks. In the following, we demonstrate that the idea of simply deleting the missing values and passing the remaining set of available data points to the LSTM cannot work in the more general case when data may be missing at intermediate time steps.

To this end, we considered the conversion reaction model (see Section 4.1) with $n_x = 11$ observations, of which we allowed at most $n_{\varnothing}^{\max} = 6$ to be absent. We trained a 5-layer cINN jointly with an LSTM with 32 hidden units on data generated according to the above strategy. Unsurprisingly, the so-trained BayesFlow network was not able to learn the correct posterior distribution, since the information on the specific position of missing observations was simply neglected. The erroneous posterior approximation for a test data set is illustrated in Fig A.

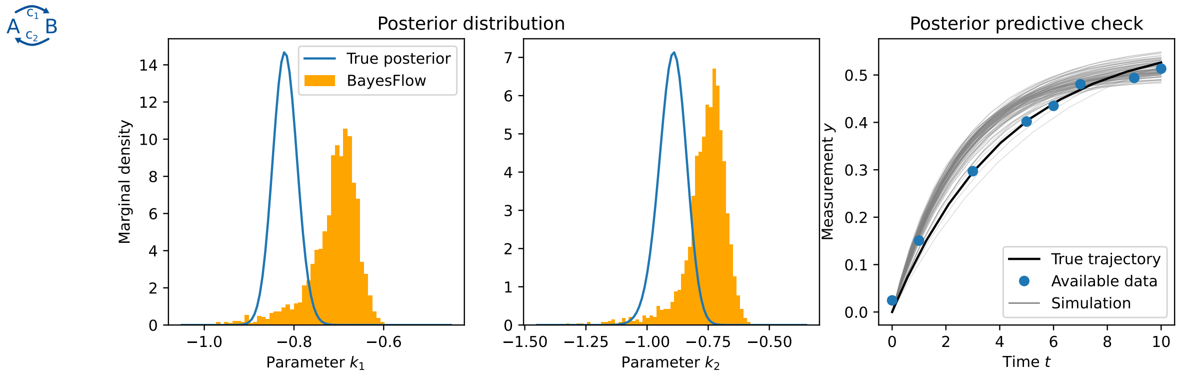

Figure A: *Simple deletion of missing data cannot work, here shown for the conversion reaction model with* $n_x = 11$ *observations.* For a test data set with missing values at $t = 2, 4, 8$, this overly simplistic approach leads to erroneous estimation of the true posterior distribution, which can also be seen from the re-simulated trajectories that fit the data poorly.

2 Linear imputation of missing data does not work

The goal of our proposed approaches is to estimate the posterior distribution $\pi(\theta|x_{\text{avai}}^{\text{obs}})$ conditioned only on the available data $x_{\text{avai}}^{\text{obs}}$. However, one might also be interested in the posterior $\pi(\theta|x^{\text{obs}})$ conditioned on the complete original data x^{obs}, since this distribution takes more information into account and is therefore more contracted. This type of inference would of course require a faithful imputation scheme $x_{\text{avai}}^{\text{obs}} \mapsto \tilde{x}^{\text{obs}}$ such that $\tilde{x}^{\text{obs}} \approx x^{\text{obs}}$.

We demonstrate that naive linear interpolation already fails for simple non-linear dynamics such as the conversion reaction model (see Section 4.1). Like in standard BayesFlow, we trained a 4-layer cINN on complete data sets. Then, for an incomplete test data set, we imputed the missing value from the available data via linear interpolation. The imputed data set was fed into the BayesFlow network trained on complete data, which resulted in misapproximated posteriors (Fig B).

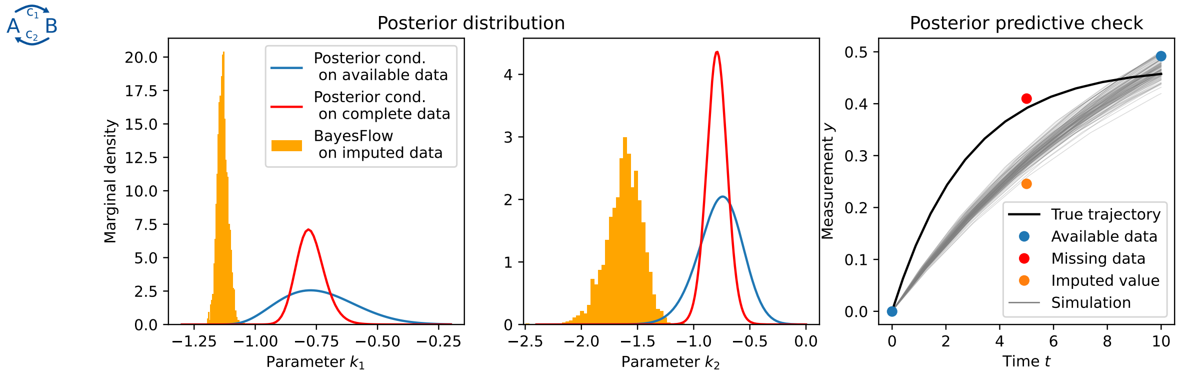

Figure B: *Naive imputation of missing data based on linear interpolation cannot work, here shown for the conversion reaction model with $n_x = 3$ observations.* Given is a test data set with a value missing at $t = 5$. The posterior samples inferred from the imputed data by the BayesFlow network trained on complete data provide a poor approximation of the desired posterior conditioned on the complete data. The reason is that the linearly interpolated value is far off the true data value. Thus, the re-simulated trajectories are forced to fit the wrongly imputed value instead of the true value of the missing observation.

3 Can imputation give more informative posteriors?

The previous section underlines the importance of a reliable imputation scheme when attempting to recover the posterior $\pi(\theta|x^{\text{obs}})$ conditioned on the complete original data. In a Bayesian setting, however, we are less interested in a one-shot estimate of the posterior $\pi(\theta|\tilde{x}^{\text{obs}})$ given one specific set of imputed data $\tilde{x}^{\text{obs}} \approx x^{\text{obs}}$, which will in general be biased, but we should rather account for the uncertainty arising from the reconstruction of missing values $x^{\text{obs}}_{\text{avai}} \mapsto \tilde{x}^{\text{obs}}$.
Concretely, assume the imputation method returns a distribution $p(x^{\text{obs}}|x^{\text{obs}}_{\text{avai}})$ of complete data sets. For each such realization x^{obs}, the amortized posterior sampling method then gives a posterior distribution $\pi(\theta|x^{\text{obs}})$. To properly account for the uncertainty in the full data x^{obs}, we need to multiply the inferred parameter probabilities $\pi(\theta|x^{\text{obs}})$ with the according data probabilities $\pi(x^{\text{obs}}|x^{\text{obs}}_{\text{avai}})$ and marginalize over all possible full data values x^{obs}.
In this, either the imputation method is biased (if available, towards additional information), or it should be a faithful approximation $p(x^{\text{obs}}|x^{\text{obs}}_{\text{avai}}) \approx \pi(x^{\text{obs}}|x^{\text{obs}}_{\text{avai}})$, where $\pi(x^{\text{obs}}|x^{\text{obs}}_{\text{avai}})\pi(x^{\text{obs}}_{\text{avai}}) = \pi(x^{\text{obs}}_{\text{avai}}, x^{\text{obs}})$ is the joint distribution of missing and complete data. That is, the imputation method recapitulates the generation of data and data missingness.
However, if we then integrate out all possible realizations of complete data, we find under the assumption $\pi(\theta|x^{\text{obs}}) = \pi(\theta|x^{\text{obs}}, x^{\text{obs}}_{\text{avai}})$:

$$\begin{aligned}\int \pi(\theta|x^{\text{obs}})p(x^{\text{obs}}|x^{\text{obs}}_{\text{avai}})\,dx^{\text{obs}} &= \int \pi(\theta|x^{\text{obs}}, x^{\text{obs}}_{\text{avai}})\pi(x^{\text{obs}}|x^{\text{obs}}_{\text{avai}})\,dx^{\text{obs}} \\ &= \int \pi(\theta, x^{\text{obs}}|x^{\text{obs}}_{\text{avai}})\,dx^{\text{obs}} \\ &= \pi(\theta|x^{\text{obs}}_{\text{avai}})\end{aligned}$$

This means we simply recover the posterior conditioned on the available data, whose estimation this paper is concerned with.
If instead $\pi(\theta|x^{\text{obs}}) \neq \pi(\theta|x^{\text{obs}}, x^{\text{obs}}_{\text{avai}})$, i.e. the missingness pattern contains information about the parameters beyond the complete data, we would still need to pass the missingness pattern to the amortized posterior sampling method, complicating its training. In doing so, we would however similarly finally obtain a marginalized posterior approximation

$$\int \pi(\theta|x^{\text{obs}}, x^{\text{obs}}_{\text{avai}})p(x^{\text{obs}}|x^{\text{obs}}_{\text{avai}})\,dx^{\text{obs}} = \pi(\theta|x^{\text{obs}}_{\text{avai}}),$$

i.e. recover the posterior given the available data (including their missingness pattern) again.
An application example in which missingness is known to be informative are clinical trials [1]. Participants might decide to drop out before a trial is concluded. The likelihood of this missingness occurring can depend on both data (e.g. when a participant gets a negative test result and subsequently loses interest to participate in follow-up tests) and model parameters (e.g. when a participant has a precondition that renders them more susceptible to side effects). Accordingly, in the latter case, the missingness pattern carries information about the parameters.

4 Model details

In this section, we introduce the mathematical models that are used to test and compare the proposed methods for encoding missing data.

4.1 Conversion reaction model

We consider the conversion process

$$X_1 \underset{c_2}{\overset{c_1}{\rightleftharpoons}} X_2$$

with rate parameters $c_1, c_2 > 0$. If we denote the concentrations of the involved chemical species by x_1 and x_2, then their dynamics can be described by the following reaction rate equations:

$$\begin{pmatrix} \dot{x}_1 \\ \dot{x}_2 \end{pmatrix} = \begin{pmatrix} -c_1 x_1 + c_2 x_2 \\ c_1 x_1 - c_2 x_2 \end{pmatrix}$$

By specifying the initial value $(x_1(0), x_2(0)) = (1, 0)$, this linear system of ordinary differential equations has the unique analytic solution:

$$\begin{pmatrix} x_1(t) \\ x_2(t) \end{pmatrix} = \frac{1}{c_1 + c_2} \left[\begin{pmatrix} c_2 \\ c_1 \end{pmatrix} + \begin{pmatrix} c_1 \\ -c_1 \end{pmatrix} e^{-(c_1+c_2)t} \right] \quad \text{for } t \geq 0$$

We assume that only the second state is measured, up to additive normal noise with known standard deviation $\sigma = 0.015$, i.e. the observation at time t is given by:

$$y_t = x_2(t) + \varepsilon_t \ \text{ with } \varepsilon_t \sim \mathcal{N}(0,\, 0.015^2) \text{ independently in } t$$

The inference is performed for the log-scale parameters $k_j = \log_{10}(c_j)$ that are believed to follow normal priors:

$$k_1, k_2 \sim \mathcal{N}(-0.75,\, 0.25^2) \ \text{ i.i.d.}$$

The prior distribution is broad enough to allow sufficiently different dynamics, but also reasonably narrow so that it is unlikely to sample model parameters leading to very flat trajectories, in which case the measurement noise would be too dominant (Fig C).
Depending on the application, we will assume, for the case of complete data, to observe data sets either consisting of $n_x = 3$ measurements at $t_0 = 0$, $t_1 = 5$ and $t_2 = 10$ or consisting of $n_x = 11$ measurements at $t = 0, 1, \ldots, 10$.

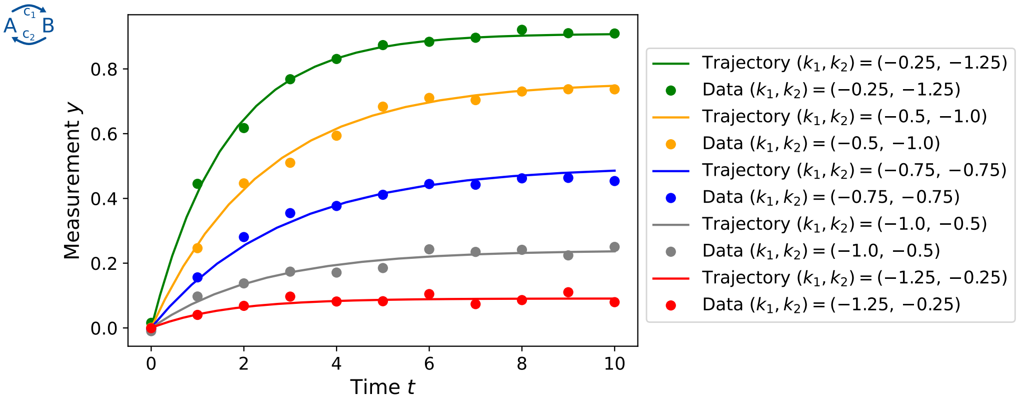

Figure C: *Conversion reaction model with $n_x = 11$ observations.* Simulation of trajectories and noisy data for different parameters (k_1, k_2) from the 2σ-interval of their respective prior distribution.

4.2 Sinusoidal model

Biochemical systems exhibiting oscillations are widely studied e.g. in the context of metabolism [2] and cell cycle [3]. From a mathematical computational perspective, dynamic models producing oscillatory data are in general hard to fit, as the landscape of the cost function to be minimized can be highly irregular and have multiple local minima [4].

As a prototype for such oscillatory models, we consider the parameterized sinusoidal function

$$x(t) = \sin(2\pi a t) + b$$

with frequency parameter a and shift parameter b. The imposed prior distributions are as follows:

$$a \sim \mathcal{U}(0.1,\, 1), \qquad b \sim \mathcal{N}(0,\, 0.25^2)$$

We assume that at time t, the value of the sine curve can be observed up to some additive normal noise:

$$y_t = x(t) + \varepsilon_t \text{ with } \varepsilon_t \sim \mathcal{N}(0,\, 0.05^2) \text{ independently in } t$$

A complete data set should contain $n_x = 41$ observations at $t_k = \frac{1}{4}k$ for $k = 0, 1, \ldots, 40$. Although seemingly harmless at first glance, this oscillatory model will already exhibit the important feature of a loss function that is difficult to optimize, so that convergence properties of different methods can be compared particularly well within the framework of this model.

4.3 FitzHugh-Nagumo model

We adapt the FitzHugh-Nagumo (FHN) model used in the studies of Pitt and Banga [4]. This is a simplified version of the Hodgkin-Huxley model [5], which describes the activation and deactivation dynamics of a spiking neuron. For us, the FHN model serves as a biological model that is capable of producing oscillatory data for certain parameter combinations.

Concretely, we consider the FHN model specified by the following non-linear system of ODEs:

$$\frac{\mathrm{d}V}{\mathrm{d}t} = \gamma \left(V - \frac{V^3}{3} + R \right),$$
$$\frac{\mathrm{d}R}{\mathrm{d}t} = -\frac{1}{\gamma}(V - \alpha + \beta \cdot R)$$

We assume uniform priors for the log-scale parameters $a = \log_{10}(\alpha)$, $b = \log_{10}(\beta)$ and $g = \log_{10}(\gamma)$:

$$a, b, g \sim \mathcal{U}(-2, 0) \text{ i.i.d.}$$

We choose the initial state $(V_0, R_0) = (-1, 1)$ and solve the initial value problem with a standard ODE solver in Python. At time t, we assume to observe the first state up to additive normal noise with known standard deviation $\sigma = 0.05$:

$$y_t = V(t) + \varepsilon_t \text{ with } \varepsilon_t \sim \mathcal{N}(0,\, 0.05^2) \text{ independently in } t$$

In the complete data case, we assume data sets consisting of $n_x = 21$ observations at $t_k = \frac{3}{4}k$ for $k = 0, 1, \ldots, 20$.

4.4 SIR ODE model

Compartmental models are nowadays a popular tool to describe and forecast the outcome of the COVID-19 pandemic [6, 7]. From a methodological viewpoint, such models are not only interesting because of their non-trivial dynamics, but also because their simulation requires at least solving an ODE.

Here, we focus on a classic SIR model [8]. Let S denote the number of susceptible, I the number of infectious and R the number of recovered individuals within a population of constant size $P = S + I + R$. Assuming the elementary reactions

$$S + I \xrightarrow{\beta} 2I, \qquad I \xrightarrow{\gamma} R \tag{1}$$

with transmission rate $\beta > 0$ and recovery rate $\gamma > 0$, the infection dynamics are quantified by the following non-linear system of ODEs:

$$\begin{aligned}\frac{\mathrm{d}S}{\mathrm{d}t} &= -\frac{\beta S I}{P},\\ \frac{\mathrm{d}I}{\mathrm{d}t} &= \frac{\beta S I}{P} - \gamma I,\\ \frac{\mathrm{d}R}{\mathrm{d}t} &= \gamma I\end{aligned}$$

We specify the population size $P = 1000$ as well as the initial state $(S_0, I_0, R_0) = (999, 1, 0)$ and solve the initial value problem employing a standard ODE solver in Python with internal error control. The data at time t are obtained by adding normally distributed measurement noise to the normalized state vector:

$$y_t = \frac{1}{P}\begin{pmatrix} S(t) \\ I(t) \\ R(t) \end{pmatrix} + \begin{pmatrix} \varepsilon_{t,1} \\ \varepsilon_{t,2} \\ \varepsilon_{t,3} \end{pmatrix} \text{ with } \varepsilon_{t,i} \sim \mathcal{N}(0,\, 0.05^2) \text{ independently in } t \text{ and } i$$

A complete data set contains $n_x = 21$ observations at $t_k = 9k$ for $k = 0, 1, \ldots, 20$. The inference is done for the log-scale parameters $b = \log_{10}(\beta)$ and $c = \log_{10}(\gamma)$ on which we impose normal priors:

$$b \sim \mathcal{N}(-1,\, 0.25^2),\ c \sim \mathcal{N}(-1.5,\, 0.25^2) \ \text{ independently}$$

4.5 SIR SSA model

To test our methods on a problem with intractable likelihood, we also consider an adapted version of the stochastic SIR model from Radev et al. [9]. Unlike the ODE model, data now come from a stochastic simulation algorithm (SSA) for the Markov jump process associated to the dynamics in (1):

$$\begin{gathered}\Delta S = -P_{SI}, \quad \Delta I = P_{SI} - P_{IR}, \quad \Delta R = P_{IR}\\ P_{SI} \sim \mathrm{Bin}\big(S, 1 - \exp(-\beta(I + 0.5)/P \cdot \Delta t)\big), \quad P_{IR} \sim \mathrm{Bin}\big(I, 1 - \exp(-\gamma \cdot \Delta t)\big)\end{gathered}$$

As stochastic simulation is often computationally expensive, an amortized approach to inference is especially desirable. We impose the following two-stage prior on the rate parameters:

$$\beta \sim \mathcal{U}(0.01,\, 1), \qquad \gamma \sim \mathcal{U}(0, \beta)$$

For the population size $P = 1000$ and the initial state $(S_0, I_0, R_0) = (999, 1, 0)$, we run the SSA for $n_{\Delta t} = 500$ time steps with step size $\Delta t = 0.1$ each. To obtain a complete data set, the normalized state vectors $y_k = \frac{1}{P}(S_k, I_k, R_k)$ at time points $t_k = \frac{5}{2}k$ for $k = 0, 1, \ldots, 20$ are collected.

4.6 Conversion reaction model with parameter-dependent missingness

In the above test problems, missing data were always sampled uniformly and independently of the underlying parameter values. This is however rarely the case in real-world applications. Therefore, we study a toy model exhibiting parameter-dependent missingness.

Concretely, we reuse the conversion reaction model (see Section 4.1) with $n_x = 11$ observations by fixing one of the rate parameters $k_2 = -0.75$ (for simplicity) and adding a missingness parameter $p \sim \mathcal{U}(0, 0.8)$ that determines the portion of missing values via $n_\varnothing = \lfloor p \cdot n_x \rfloor$. More specifically, this means that $n_\varnothing$ values of the n_x are sampled uniformly and set to be missing (see Fig D). Given simulated data from this model, we seek to estimate the joint posterior of k_1 and p. Special attention is paid to whether the following can be recovered: The independence of the dynamics parameter k_1 from the missingness parameter p, as well as the uniform marginal distribution of p reflecting the interval of parameter values that lead to the observed number of missing data.

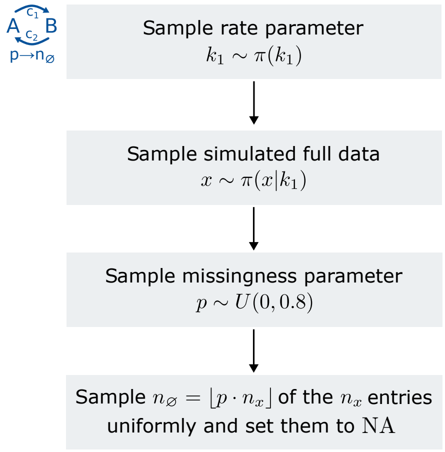

Figure D: *Conversion reaction model with parameter-dependent missingness.* Visualization of the data generating process, where missingness sampling depends on a model parameter p.

5 Supplementary error analysis

In this section, we provide additional plots to illustrate the performance of our methods on the test problems.

5.1 Methods

First, we briefly introduce the employed performance validation techniques.

- **Validation metrics**: The normalized root mean squared error NRMSE and the coefficient of determination R^2 are defined as follows for a sample of true parameters $\{\nu^{(m)}\}_{m=1}^M$ and a sample of estimated parameters $\{\widehat{\nu}^{(m)}\}_{m=1}^M$:

$$\mathrm{NRMSE} := \sqrt{\sum_{m=1}^{M} \frac{\left(\nu^{(m)} - \widehat{\nu}^{(m)}\right)^2}{\nu_{\max} - \nu_{\min}}}, \qquad R^2 := 1 - \frac{\sum_{m=1}^{M} \left(\nu^{(m)} - \widehat{\nu}^{(m)}\right)^2}{\sum_{m=1}^{M} \left(\nu^{(m)} - \bar{\nu}\right)^2}$$

  Here, $\nu_{\max}$, $\nu_{\min}$ and $\bar{\nu}$ denote the maximum, minimum and mean of the true parameters, respectively. NRMSE measures how accurately the true parameter values are recovered by the estimates, and R^2 measures the proportion of variation in the sample of true parameters that is explained by the sample of estimated parameters. Perfect recovery is achieved when NRMSE $= 0$ and $R^2 = 1$.

  In our experiments, we chose $M = 500$, i.e. we computed the metrics on the basis of 500 test data sets. For the conversion reaction model, we compared the empirical means of the "true" posteriors obtained via MCMC sampling (using the emcee tool, Foreman-Mackey et al. [10]), with the empirical means of the estimated posteriors from BayesFlow. For the sinusoidal model, we compared the ground truth parameters (data generating parameters) with the empirical means of the estimated posteriors. This is justified as we consider a relatively high number of observations in the sinusoidal model, even in the case of missing data, such that the ground truth parameters will give a good approximation of the true posterior means.

- **Simulation-based calibration**: Simulation-based calibration (SBC) exploits the insight that the Bayesian joint distribution is self-consistent [11]. Concretely, it can be shown that averaging the exact posterior $\pi(\theta|x')$ over data x' coming from the joint distribution $\pi(\theta', x') = \pi(\theta')\pi(x'|\theta')$ will recover the prior distribution (for a proof, see Appendix B in Radev et al. [9]):

$$\pi(\theta) = \iint \pi(\theta|x')\pi(\theta', x')\, d\theta' dx'$$

  If the exact posterior is replaced by an inadequate approximation, the above equality will be violated. Such violations can be detected by computing rank statistics which compare a sample of data generating prior parameters with the according approximate posterior samples and inspecting the resulting histogram for uniformity (SBC plot). While uniform SBC plots indicate good approximation, different types of deviations from the uniformity allow interpretations such as over-/underfitting or a systematic bias in the approximate posteriors. For illustrative examples of such deviations and their interpretations, we refer to Talts et al. [11].

  In our test problems, we computed the SBC histograms using 5000 prior parameters (5000 data sets) and 250 posterior samples per data set.

5.2 Conversion reaction model

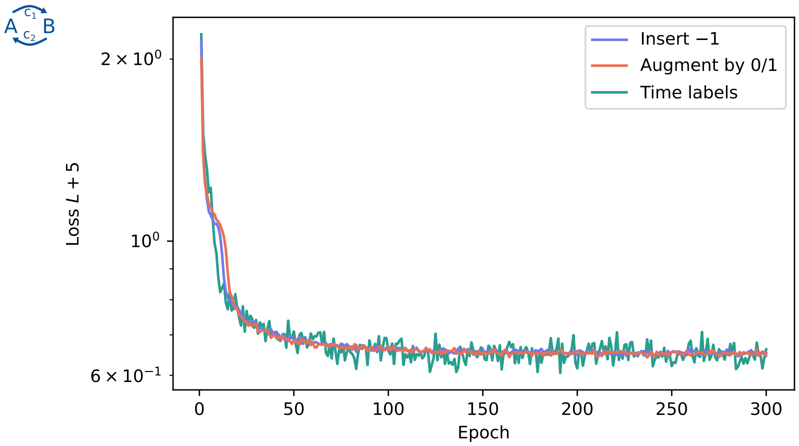

Figure E: *Convergence plot for the conversion reaction model with* $n_x = 3$ *observations.* For this simple test problem, all three proposed approaches result in a similar convergence rate of the loss function. The loss curve for the “Time labels” approach is more jumpy due to the sampling of missingness on the batch level.

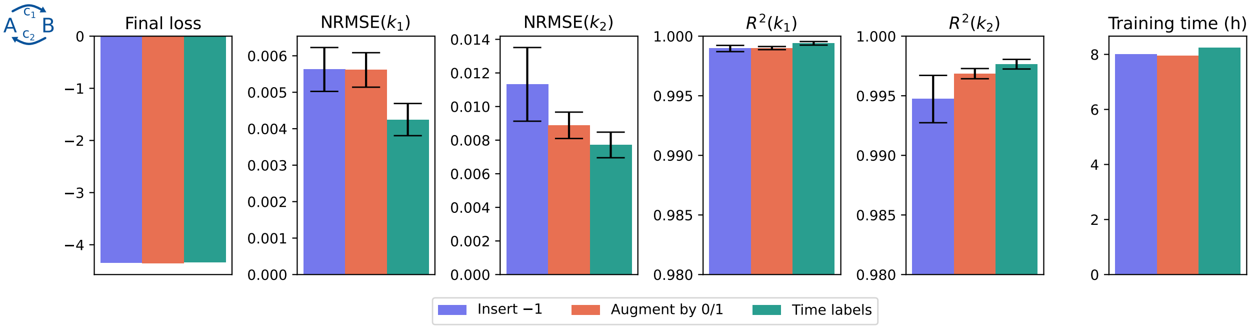

Figure F: *Error metrics for the conversion reaction model with* $n_x = 3$ *observations.* The three encodings lead to similar error metrics in terms of the final loss as well as NRMSE and R^2 scores. The “Time labels” approach performed marginally superior on this problem. The training time is approximately the same for all three encodings.

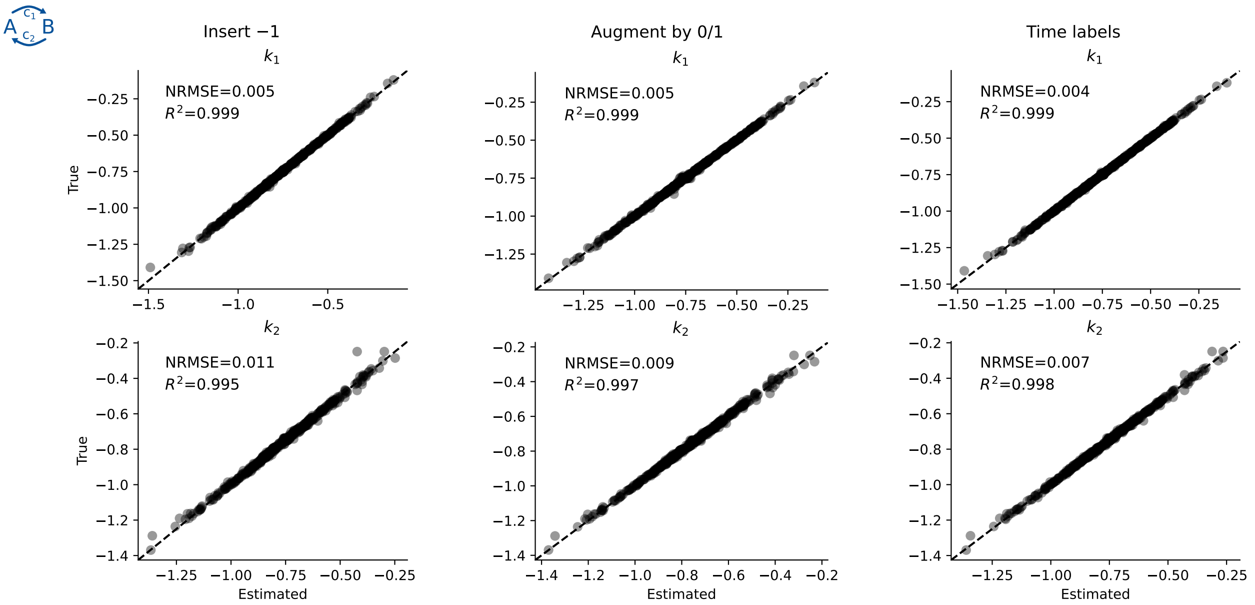

Figure G: *True vs. estimated for the conversion reaction model with* $n_x = 3$ *observations.* Posterior means estimated by the BayesFlow network are in great accordance with the “true” means obtained via MCMC sampling (using the emcee tool, Foreman-Mackey et al. [10]), showing that all three encodings perform very well on this test problem. Marginal differences as in Fig F result from a tiny portion of data sets.

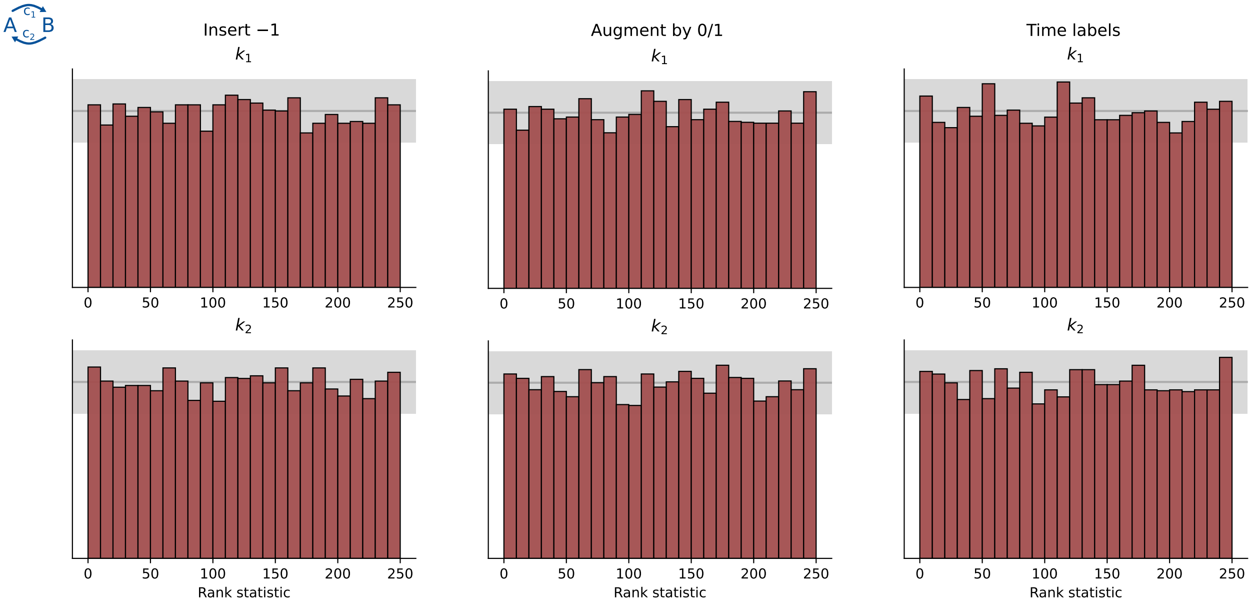

Figure H: *SBC for the conversion reaction model with* $n_x = 3$ *observations.* The histograms exhibit uniformity, indicating the networks have converged and no systematic bias or over-/underfitting of the posteriors is detected.

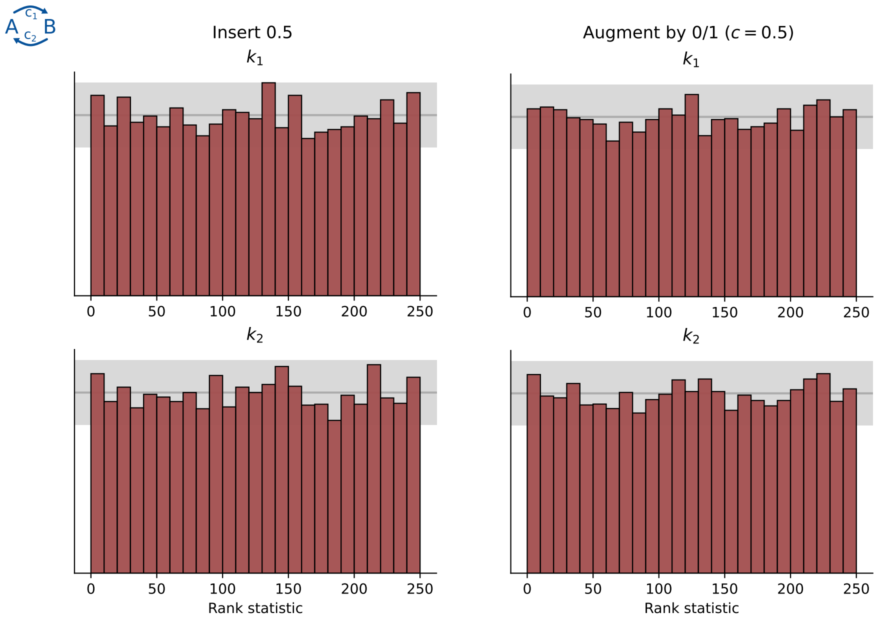

Figure I: *SBC for the conversion reaction model with $n_x = 3$ observations (investigating ambiguous dummy imputation values).* No systematic bias or over-/underfitting of the posteriors is detected for the trained networks.

5.3 Sinusoidal model

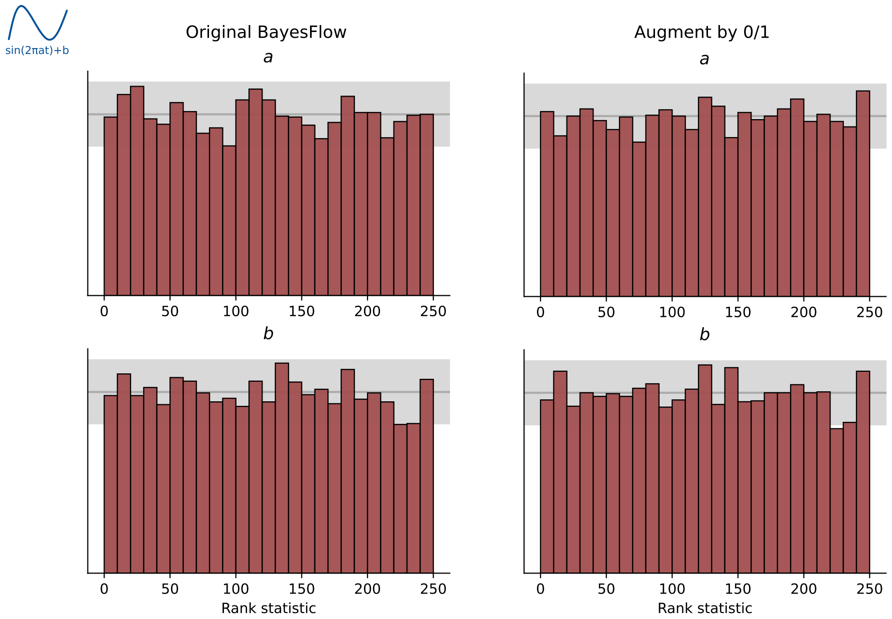

Figure J: *SBC for the sinusoidal model with variable data set length.* No systematic bias or over-/underfitting in the posteriors is captured by the histograms.

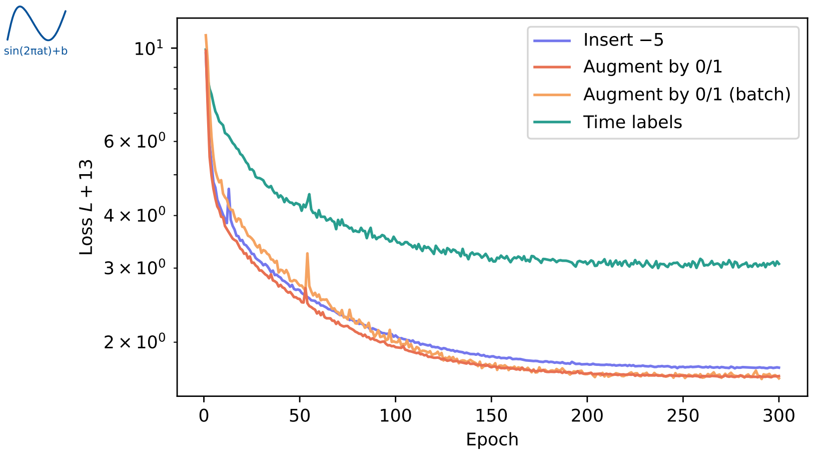

Figure K: *Convergence plot for the sinusoidal model with uniformly sampled missing time steps.* The approach "Augment by 0/1" performs the most robustly: It achieves a lower final loss than "Insert −5" although $c = -5$ is an unambiguous dummy value for this model. The approach "Time labels" converges very poorly. This behavior cannot be merely explained by the sampling of number of missing observations on batch level, as it cannot be reproduced by using the binary augmentation with batch sampling ("Augment by 0/1 (batch)").

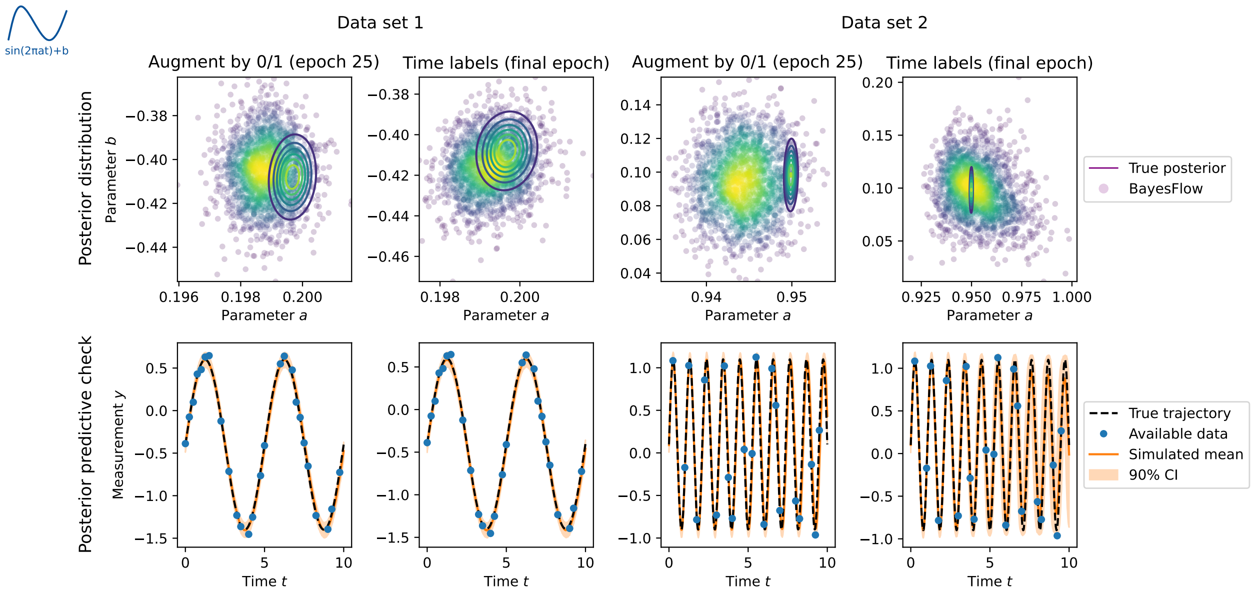

Figure L: *Posterior estimation and predictive check for the sinusoidal model with uniformly sampled missing time steps, comparing "Time labels" and "Augment by 0/1" in an earlier generation.* The final loss achieved by the network trained with the encoding "Time labels" is approximately reached by the network trained with "Augment by 0/1" already in epoch 25. Here, we compare the posterior approximation of "Augment by 0/1" at this early stage of training with the final posterior approximation obtained by "Time labels". We observe similarly poor approximations of the posteriors.

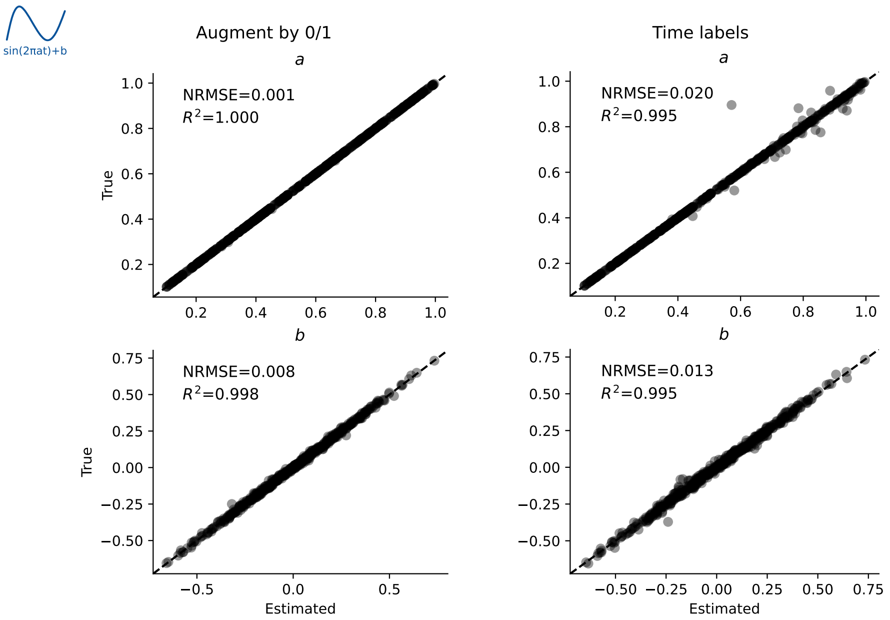

Figure M: *True vs. estimated for the sinusoidal model with uniformly sampled missing time steps.* The encoding "Time labels" leads to larger deviations of the estimated means from the ground truth than the encoding "Augment by 0/1".

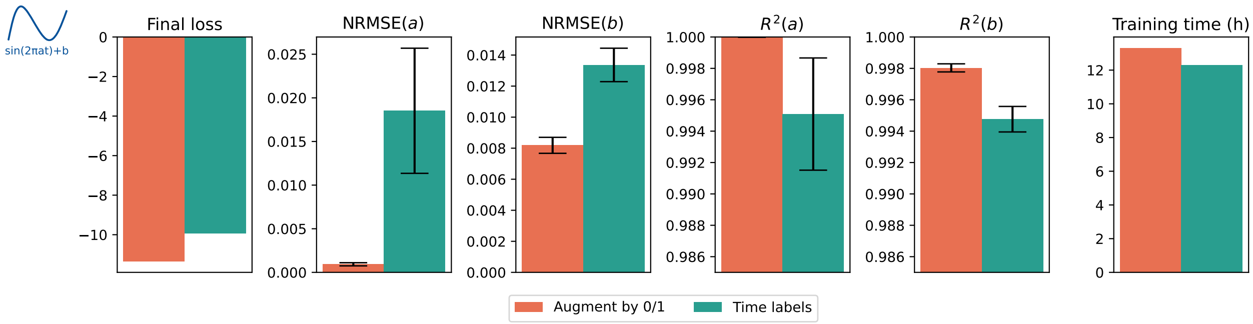

Figure N: *Error metrics for the sinusoidal model with uniformly sampled missing time steps.* In accordance with Fig M, the error metrics NRMSE and R^2 indicate a much better approximation of the ground truth parameters by the approach "Augment by 0/1" than by "Time labels".

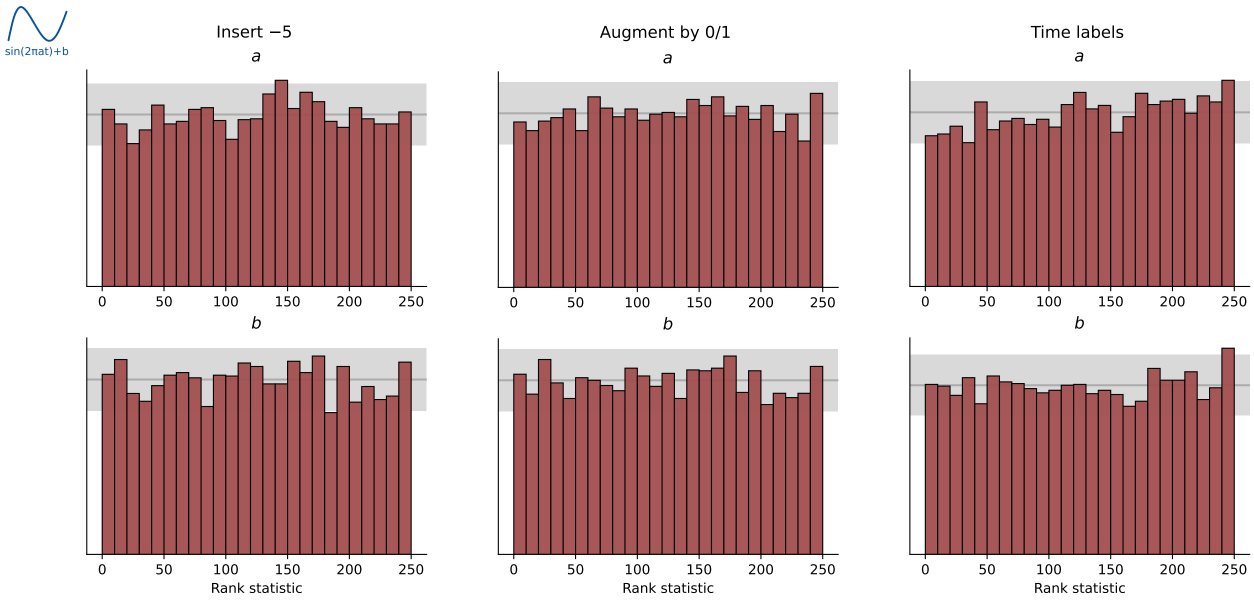

Figure O: *SBC for the sinusoidal model with uniformly sampled missing time steps.* No clear systematic bias or over-/underfitting in the posteriors is detected by the histograms.

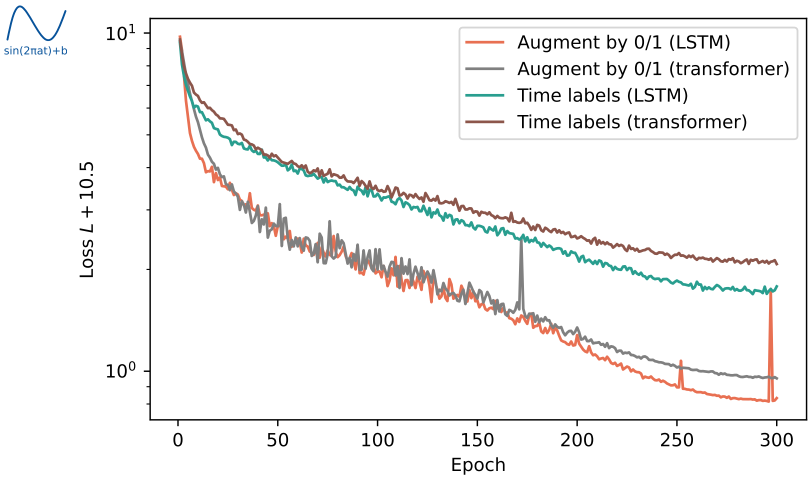

Figure P: *Convergence plot for the sinusoidal model with uniformly sampled missing time steps, comparing LSTM and transformer summary networks.* Due to bad convergence of the "Time labels" approach, we thought about alternative architectures for the summary network. A reasonable alternative are attention-based transformer networks that are able to extract features from time series data with positional encodings. As such network is implemented in BayesFlow version 1.1, we used this version for the following analysis, along with standard network and training hyperparameters. We compared the performance of the encodings "Augment by 0/1" and "Time labels", each in combination with the usual LSTM as well as the transformer summary network. We observe that "Augment by 0/1" together with an LSTM still converges the best and that in particular, the performance of "Time labels" is not improved by using the alternative summary network architecture.

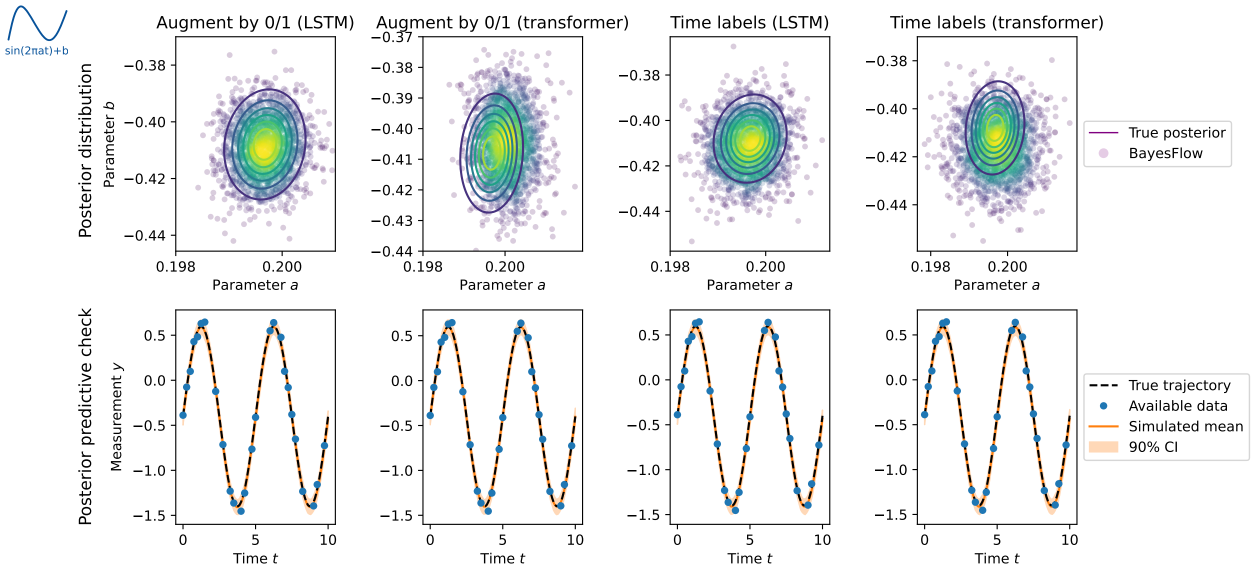

(a) Posterior samples and predictive checks for Data set 1 (Parameters $[0.2, -0.4]$, $n_\varnothing = 15$)

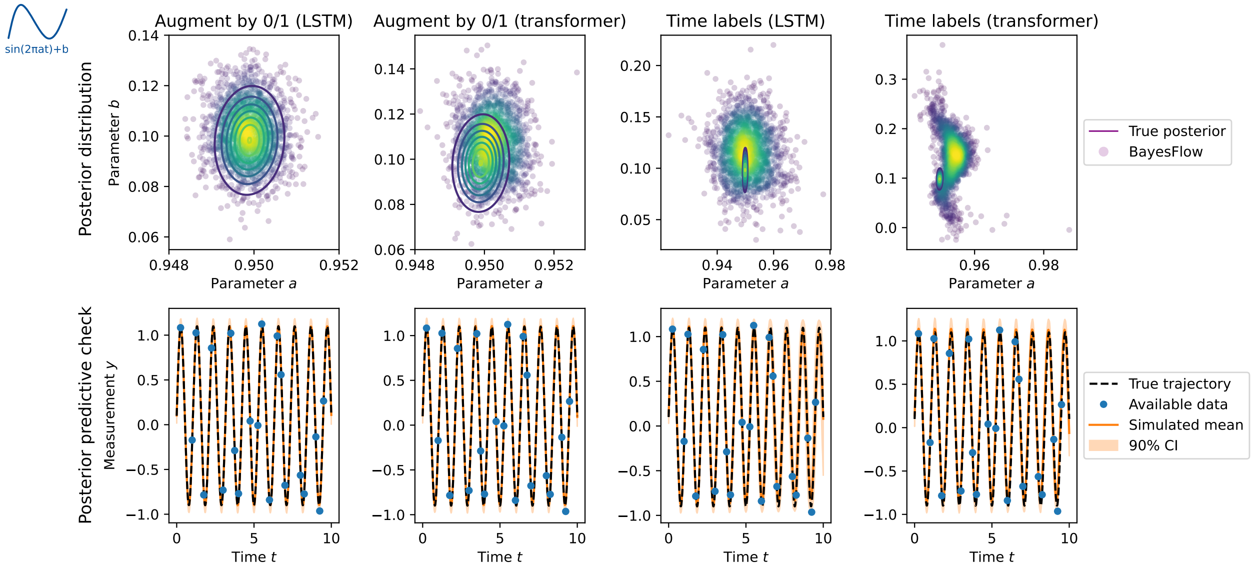

(b) Posterior samples and predictive checks for Data set 2 (Parameters $[0.95, 0.1]$, $n_\varnothing = 20$)

Figure Q: *Results for the sinusoidal model with uniformly sampled missing time steps, comparing LSTM and transformer summary networks.* Respective top: Posterior distributions. Respective bottom: Posterior predictive checks showing the means of noise-corrupted simulations and their centered 90% credible intervals. In accordance with Fig P, we observe "Augment by 0/1" together with the usual LSTM summary network yields the best posterior approximations, whereas the already poor performance of "Time labels" is even worsened when changing over to the transformer summary network.

5.4 FitzHugh-Nagumo model

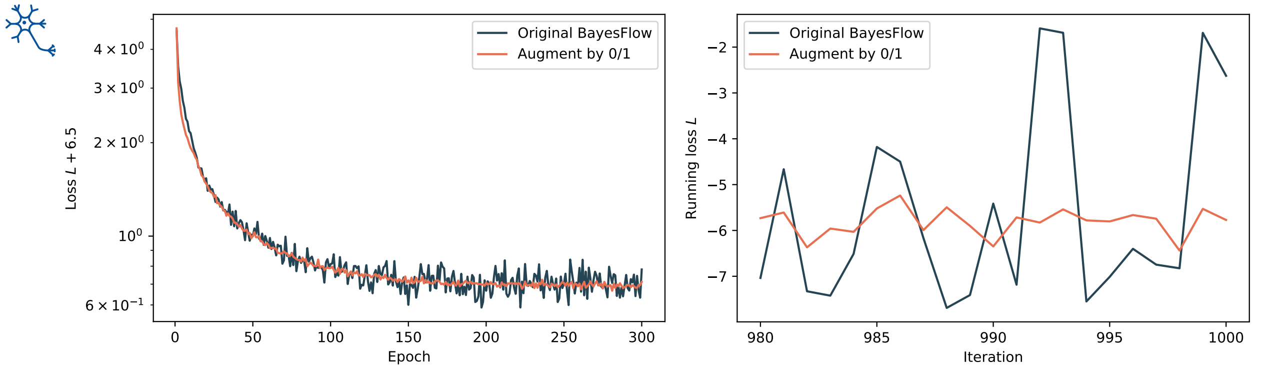

(a) Epoch-averaged loss over all 300 training epochs. (b) Loss in the last 20 iterations of the final epoch.

Figure R: *Comparison of loss behavior for the FHN model with variable data set length.* The loss of the original BayesFlow method exhibits much stronger fluctuations (b) than "Augment by 0/1" due to batch sampling the data set length. Unlike for the sinusoidal model, the overall convergence (a) is however not negatively affected.

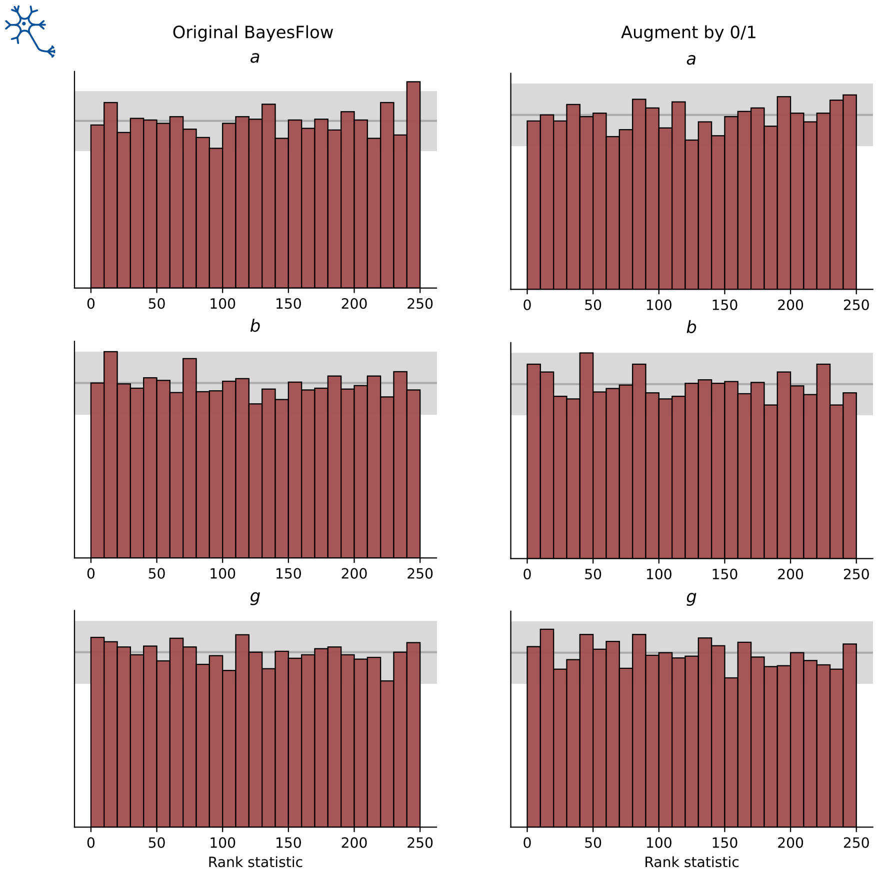

Figure S: *SBC for the FHN model with variable data set length.* The histograms indicate no systematic bias or over-/underfitting in the posteriors.

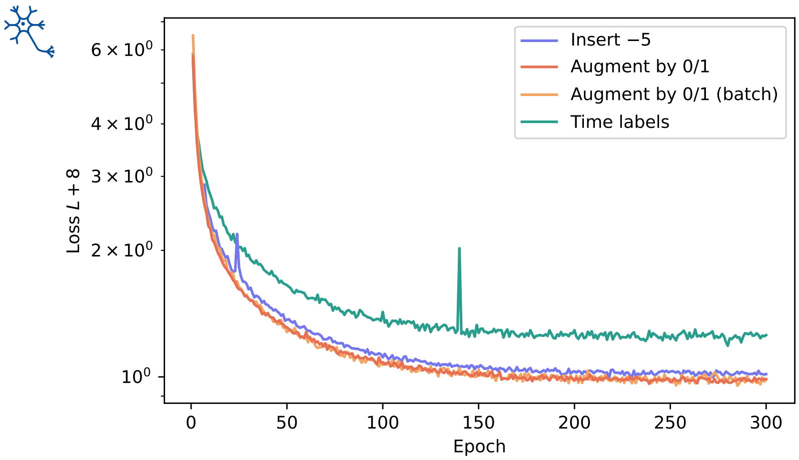

Figure T: *Convergence plot for the FHN model with uniformly sampled missing time steps.* The approach "Augment by 0/1" performs the most robustly: It achieves a lower final loss than "Insert -5", although $c = -5$ is an unambiguous dummy value for this model. The approach "Time labels" converges very poorly. This behavior cannot be merely explained by the sampling of number of missing observations on batch level, as it cannot be reproduced by using the binary augmentation with batch sampling ("Augment by 0/1 (batch)"). These results are very similar to the ones from the sinusoidal model – it seems that in the case of oscillatory data, the network may misinterpret the time labels and thus not converge properly.

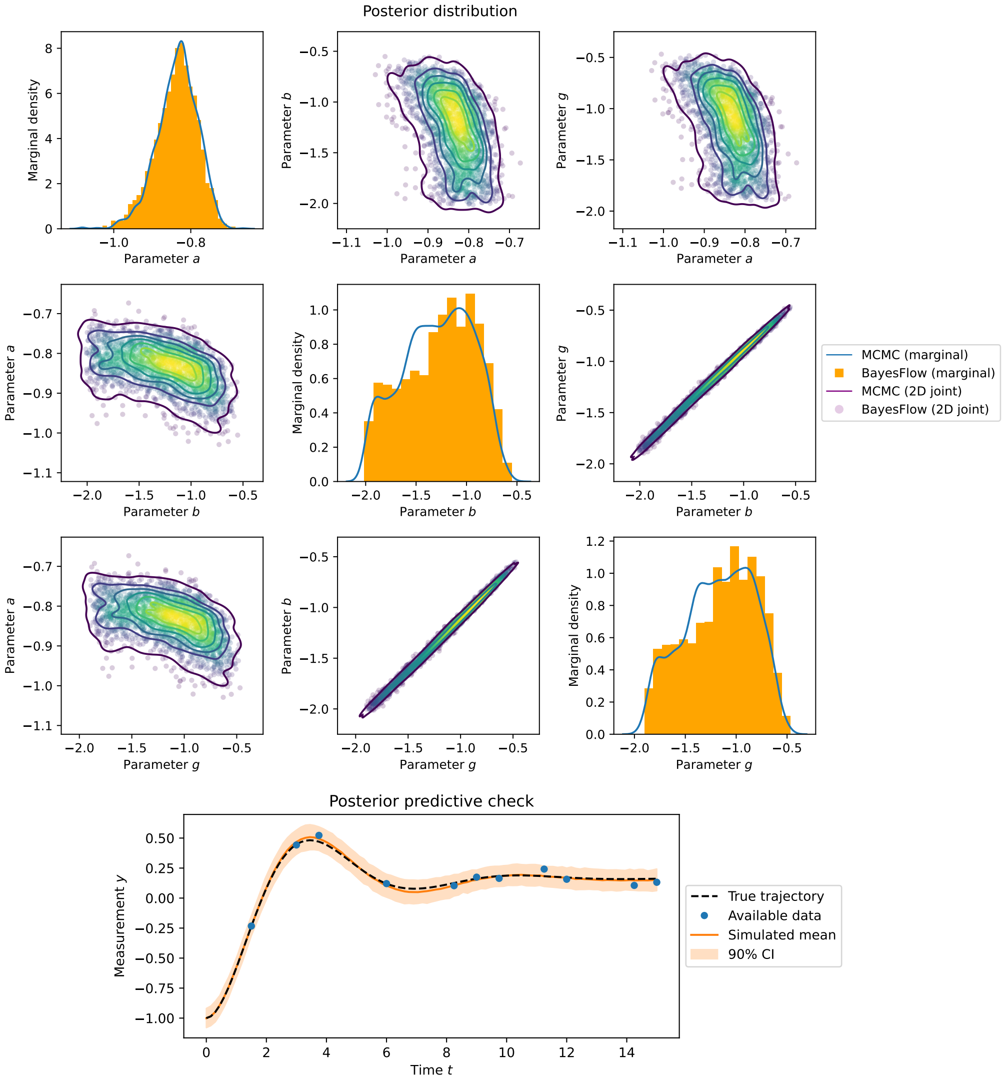

(a) Posterior samples and predictive check for Data set 1 using "Augment by 0/1"

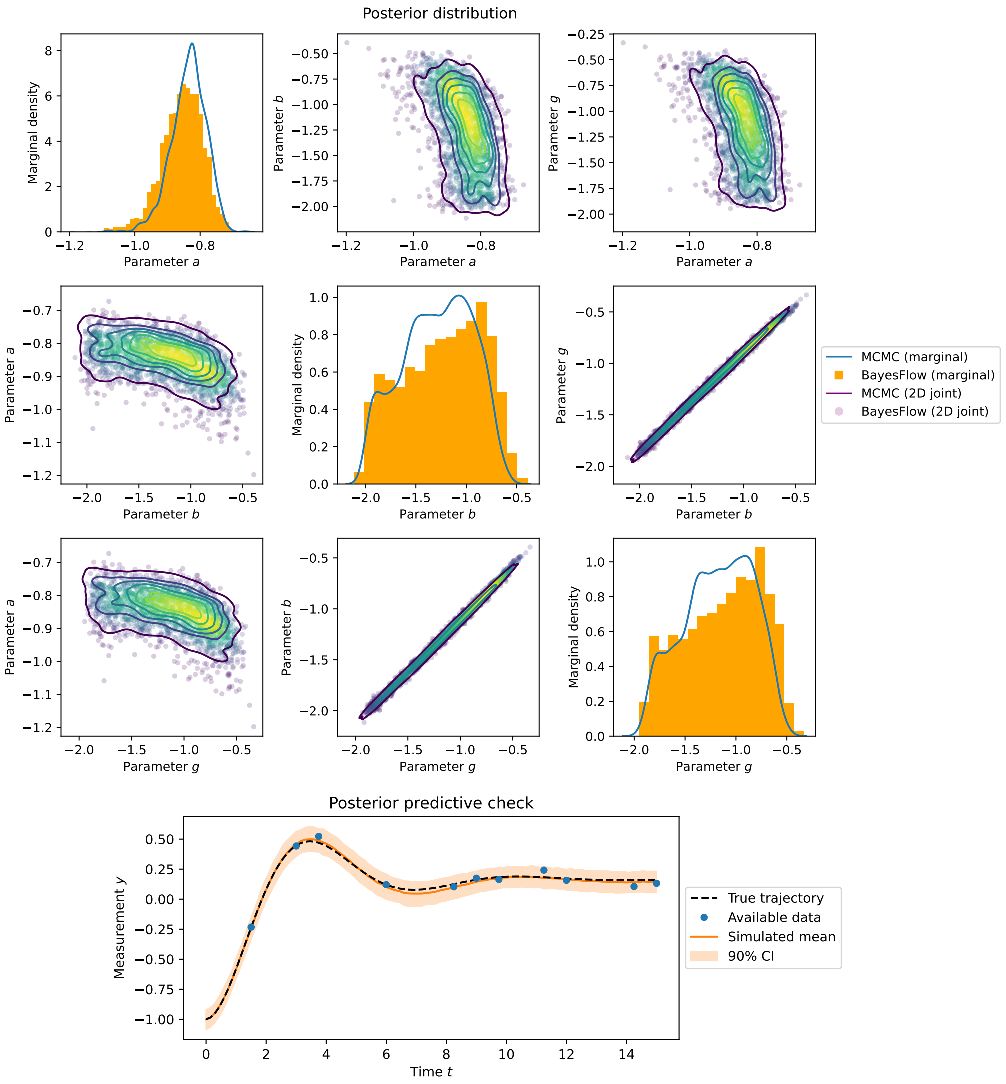

(b) Posterior samples and predictive check for Data set 1 using “Time labels”

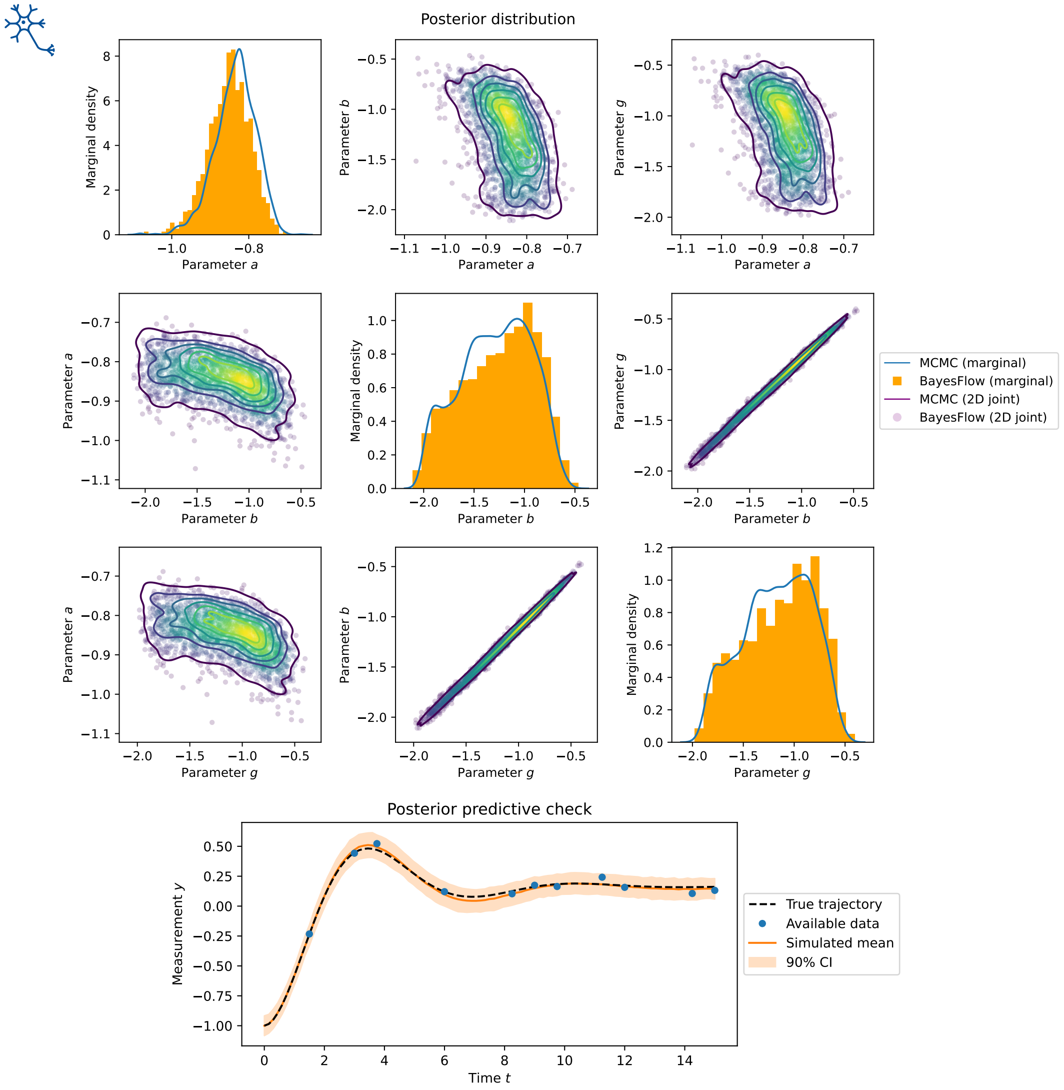

(c) Posterior samples and predictive check for Data set 1 using "Augment by 0/1" in an earlier generation

Figure U: *Results for the FHN model with uniformly sampled missing time steps, Data set 1 (at parameters* $[-0.8, -1.4, -1.3]$*,* $n_{\varnothing} = 10$*), comparing the encodings (a) "Augment by* $0/1$*", (b) "Time labels" and (c) "Augment by* $0/1$*" in an earlier generation.* Respective top: Posterior distributions. Respective bottom: Posterior predictive checks showing the means of noise-corrupted simulations and their centered 90% credible intervals. We observe that "Augment by 0/1" yields slightly better posterior approximation.

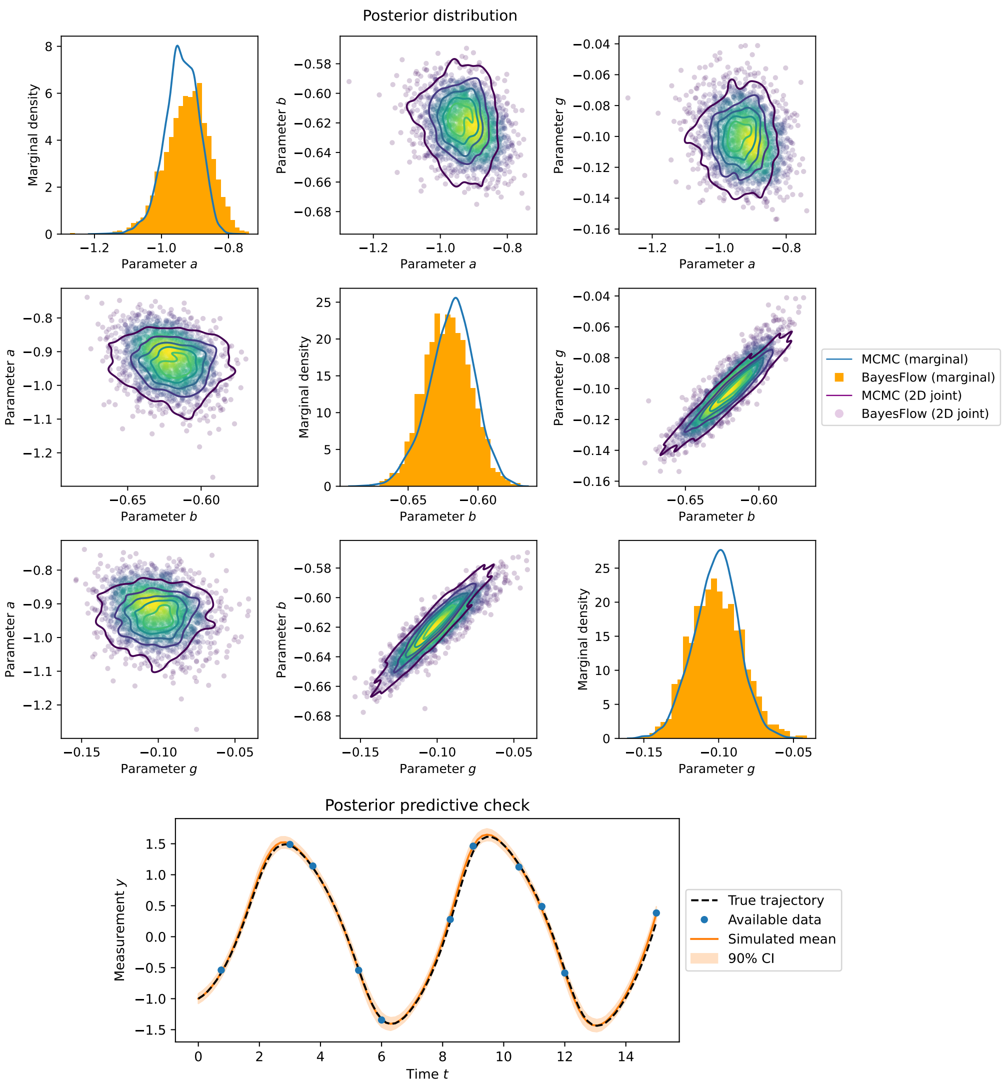

(a) Posterior samples and predictive check for Data set 2 using "Augment by 0/1"

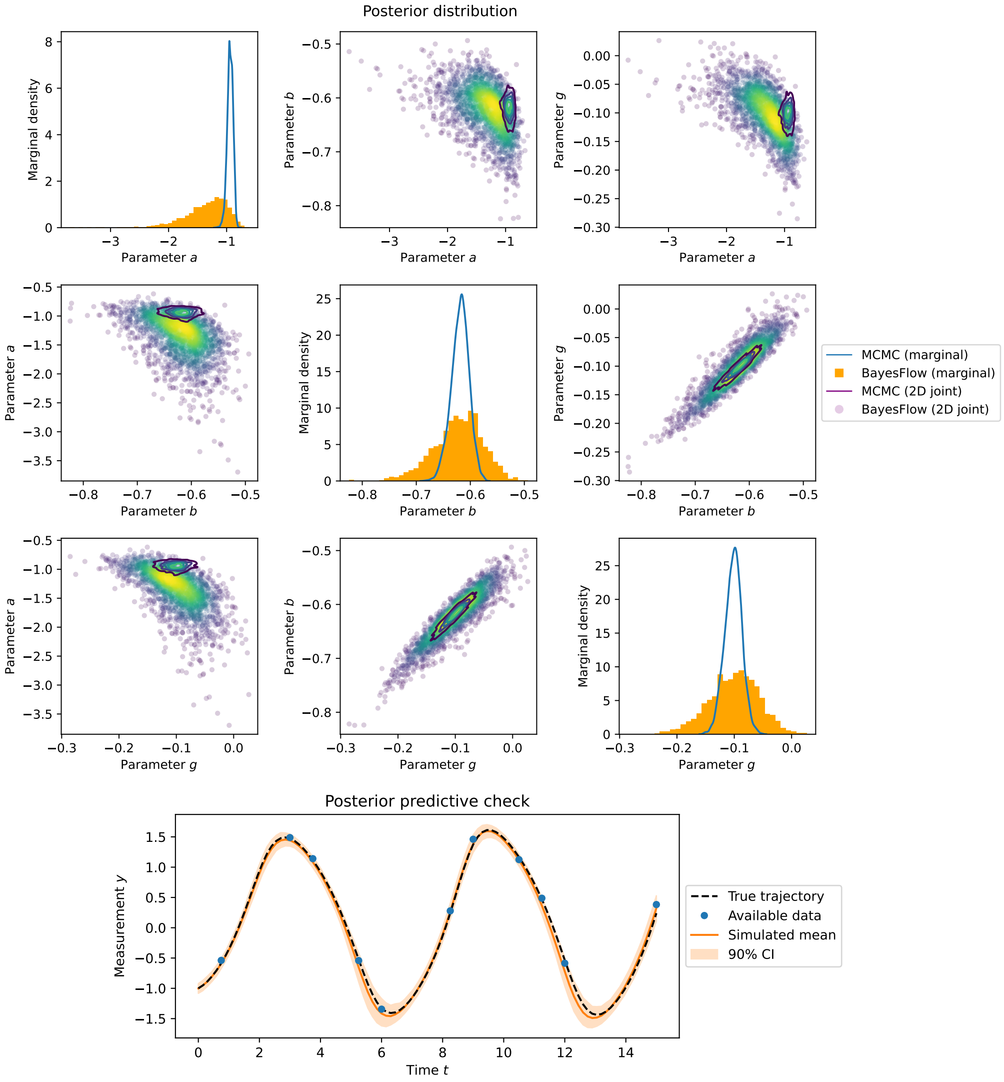

(b) Posterior samples and predictive check for Data set 2 using “Time labels”

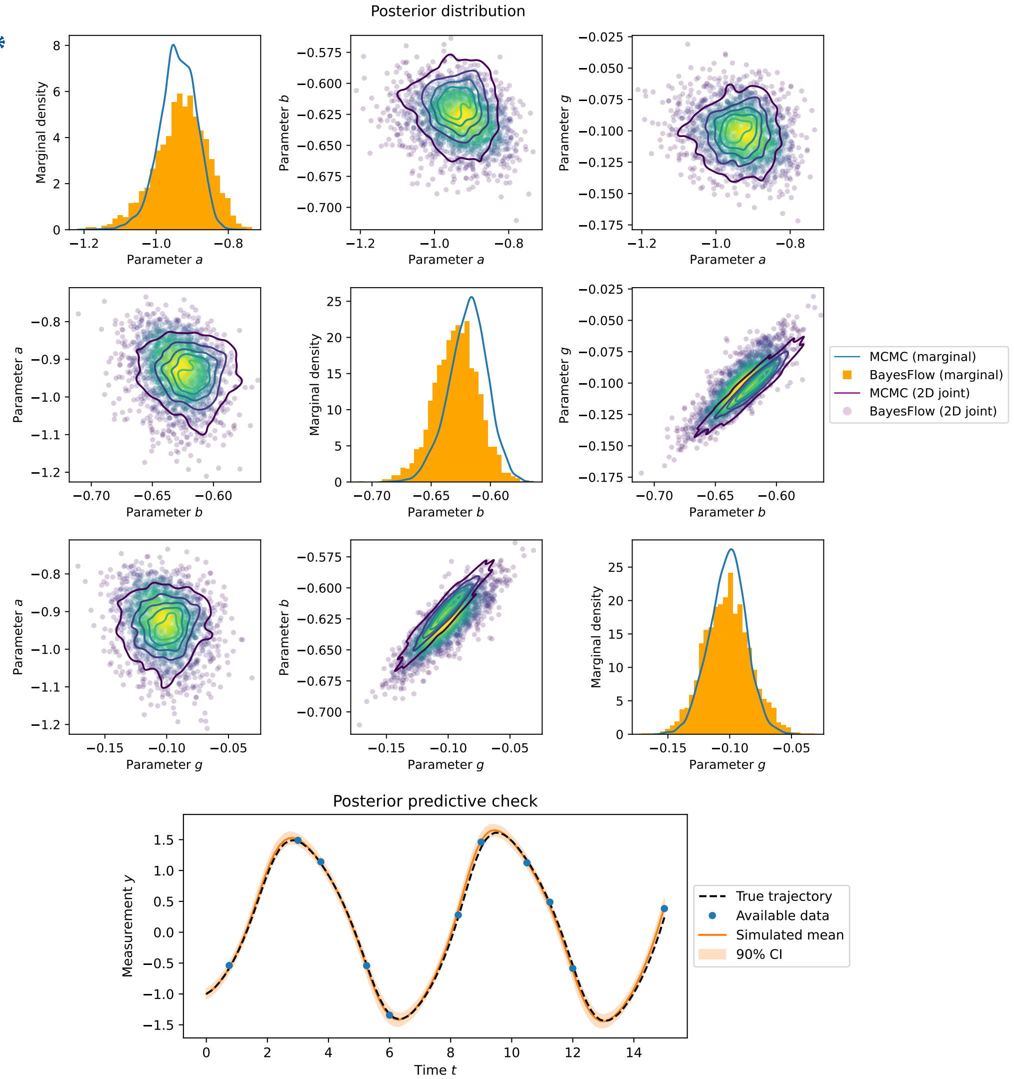

(c) Posterior samples and predictive check for Data set 2 using “Augment by 0/1” in an earlier generation

Figure V: *Results for the FHN model with uniformly sampled missing time steps, Data set 2 (at parameters* $[-1.0, -0.6, -0.1]$*,* $n_{\varnothing} = 10$*), comparing the encodings (a) “Augment by* $0/1$*”, (b) “Time labels” and (c) “Augment by* $0/1$*” in an earlier generation.* Respective top: Posterior distributions. Respective bottom: Posterior predictive checks showing the means of noise-corrupted simulations and their centered 90% credible intervals. We observe that “Time labels” performs poorly. In contrast, “Augment by 0/1”, which reaches the final loss function value of “Time labels” in training epoch 56, already performs decently in that epoch and improves again slightly upon finishing network training.

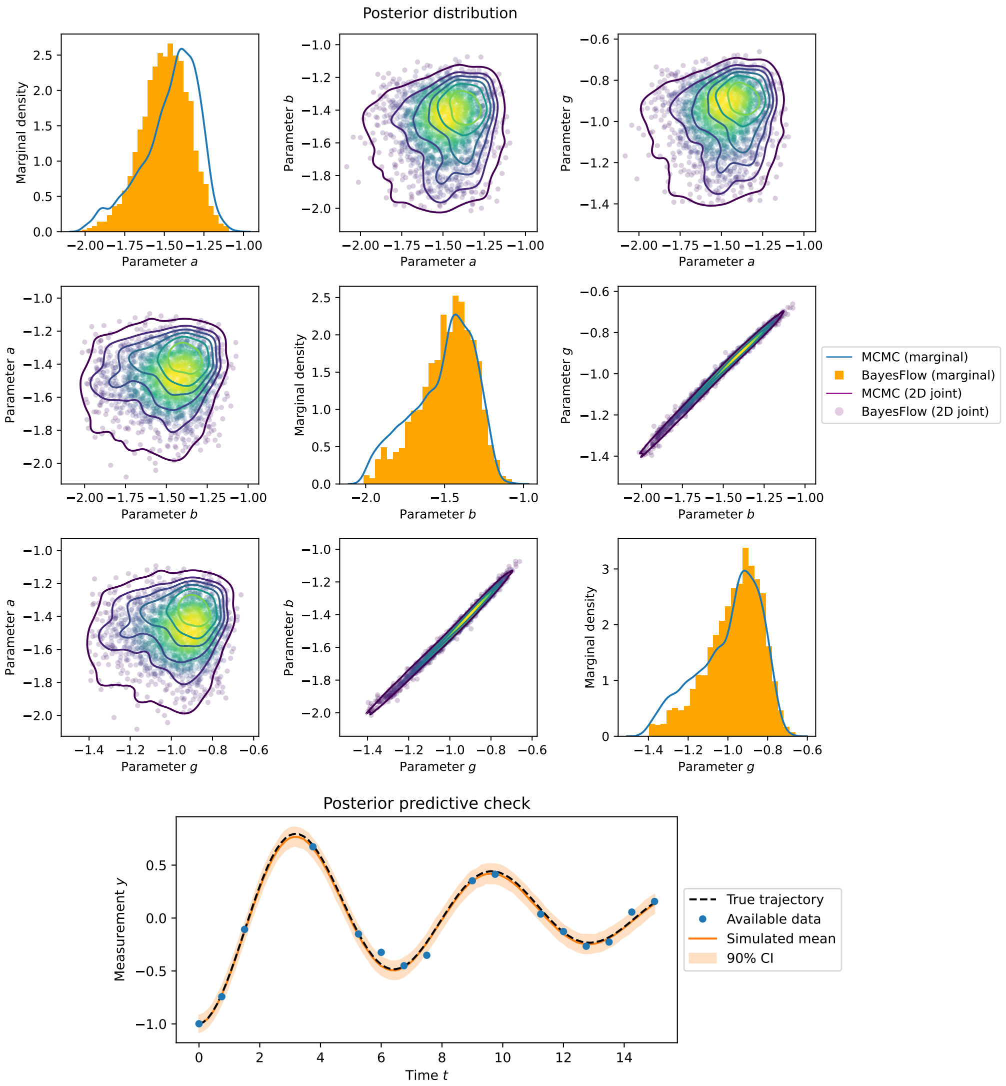

(a) Posterior samples and predictive check for Data set 3 using “Augment by 0/1”

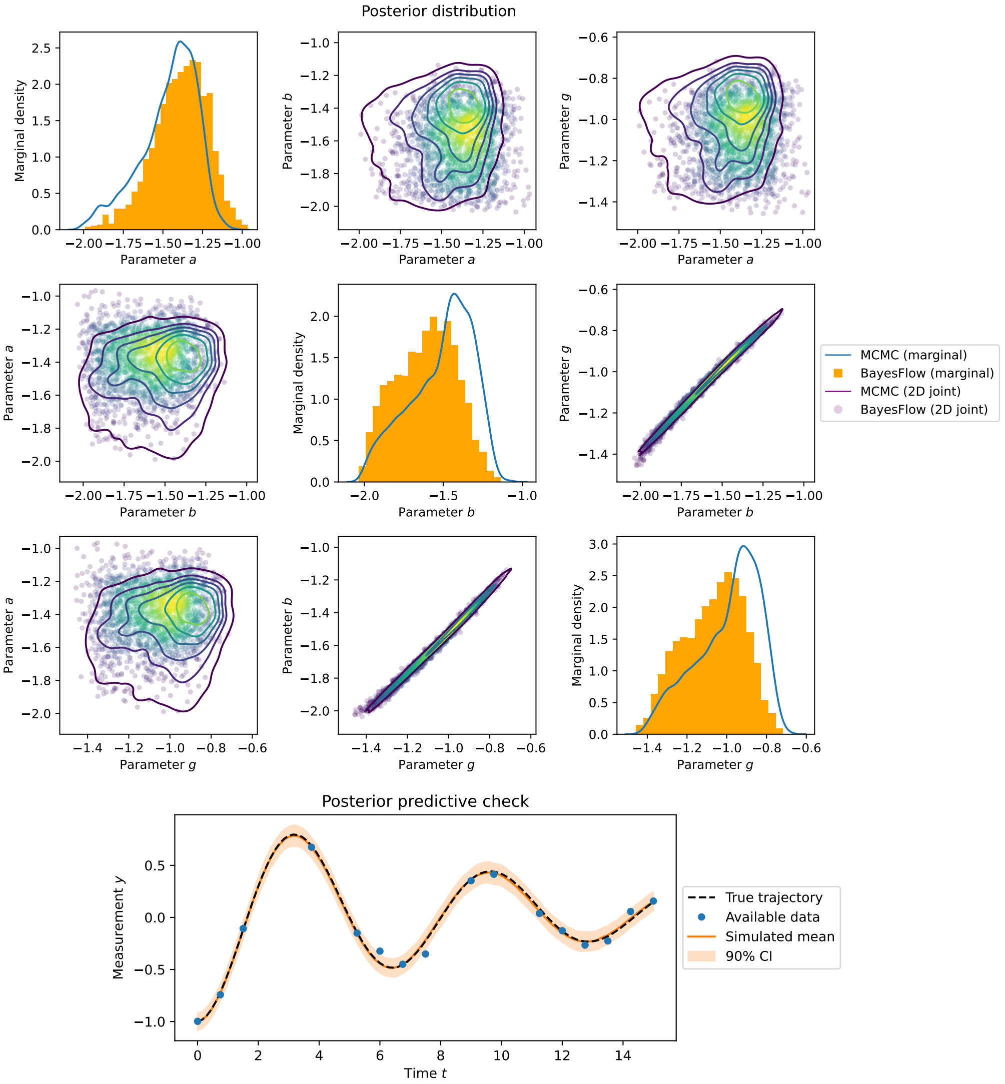

(b) Posterior samples and predictive check for Data set 3 using “Time labels”

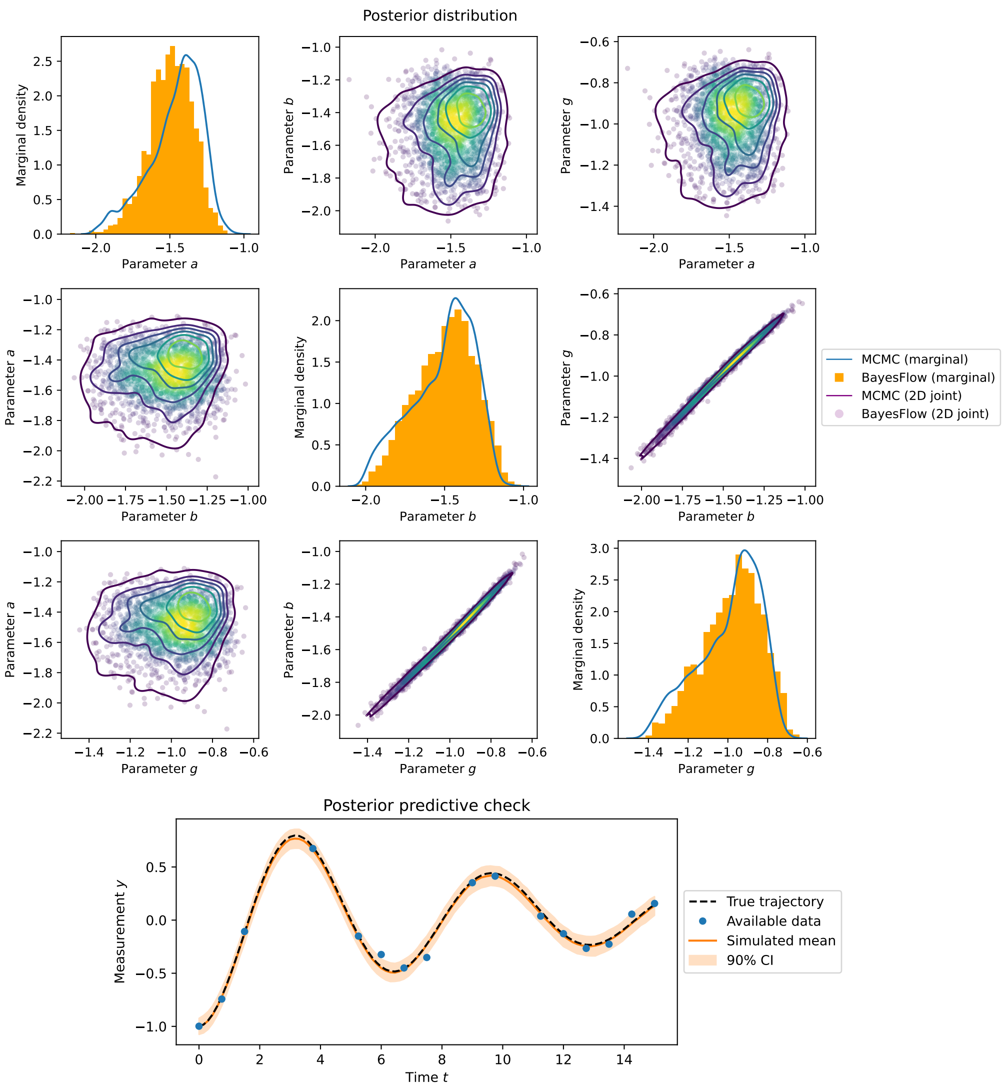

(c) Posterior samples and predictive check for Data set 3 using “Augment by 0/1” in an earlier generation

Figure W: *Results for the FHN model with uniformly sampled missing time steps, Data set 3 (at parameters* $[-1.3, -1.4, -0.9]$, $n_{\varnothing} = 5$*), comparing the encodings (a) “Augment by* $0/1$*”, (b) “Time labels” and (c) “Augment by* $0/1$*” in an earlier generation.* Respective top: Posterior distributions. Respective bottom: Posterior predictive checks showing the means of noise-corrupted simulations and their centered 90% credible intervals. We observe that “Augment by 0/1” yields better posterior approximation.

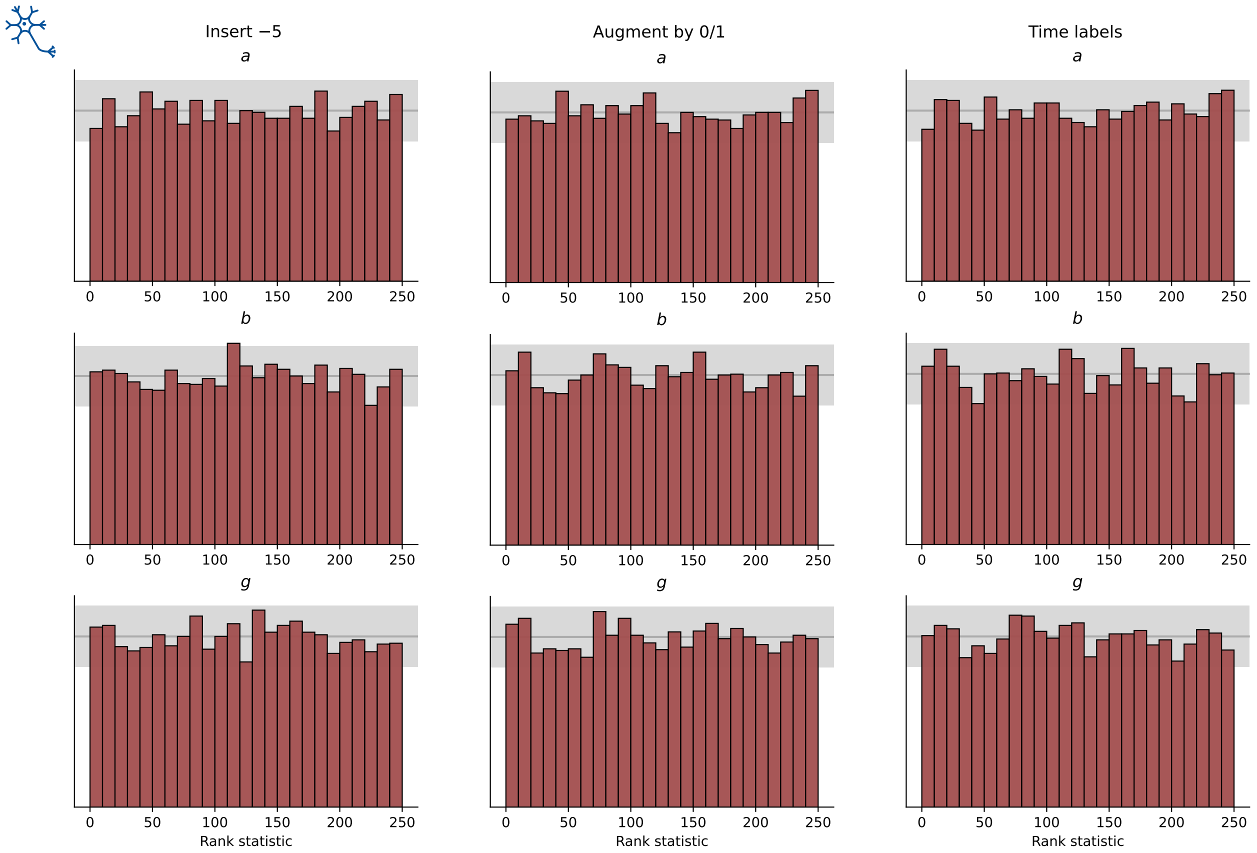

Figure X: *SBC for the FHN model with uniformly sampled missing time steps.* For all three encodings, the histograms capture no systematic bias or over-/underfitting in the posteriors.

5.5 SIR ODE model

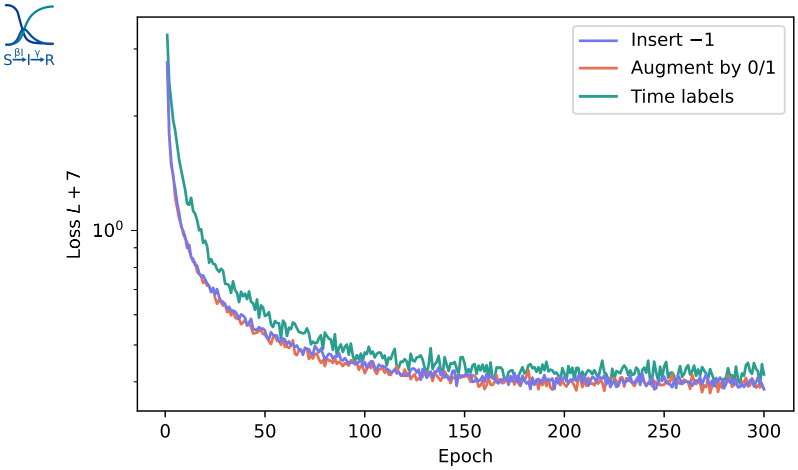

Figure Y: *Convergence plot for the SIR ODE model.* The losses of the networks trained with the encodings “Augment by 0/1” and “Insert −1” behave similarly. They converge faster, more smoothly and towards a lower final value than the loss for “Time labels”. Due to the rather large value of $t_{\text{end}} = 180$, we used normalized time points as positional encoding for the “Time labels” approach.

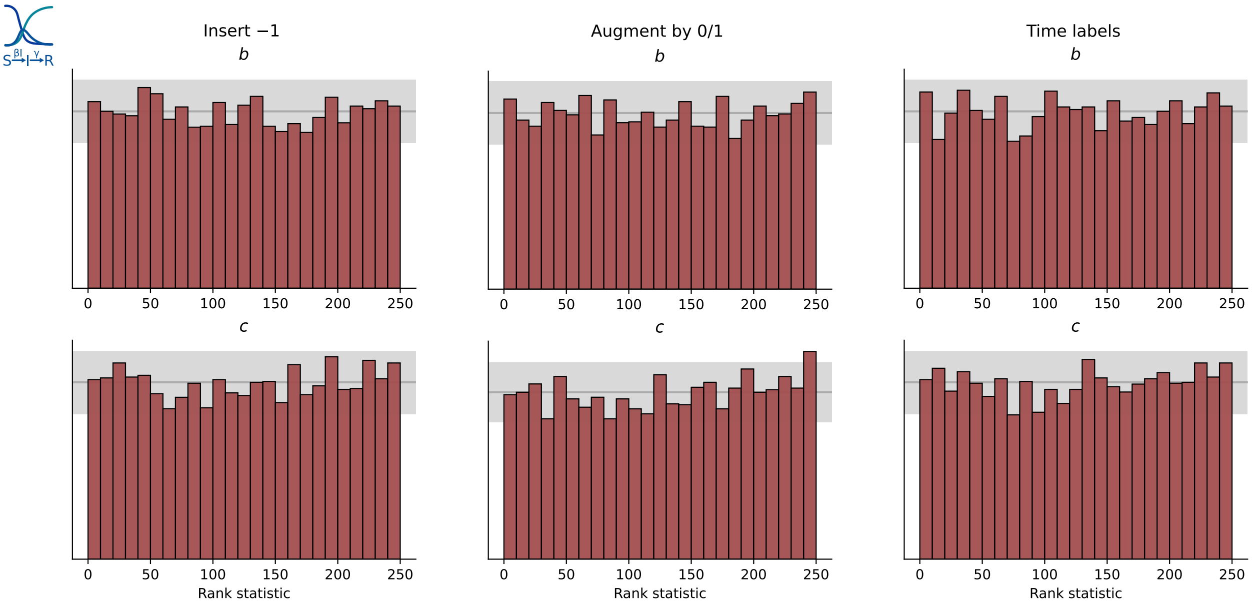

Figure Z: *SBC for the SIR ODE model.* No clear systematic bias or over-/underfitting in the posteriors is detected by the histograms.

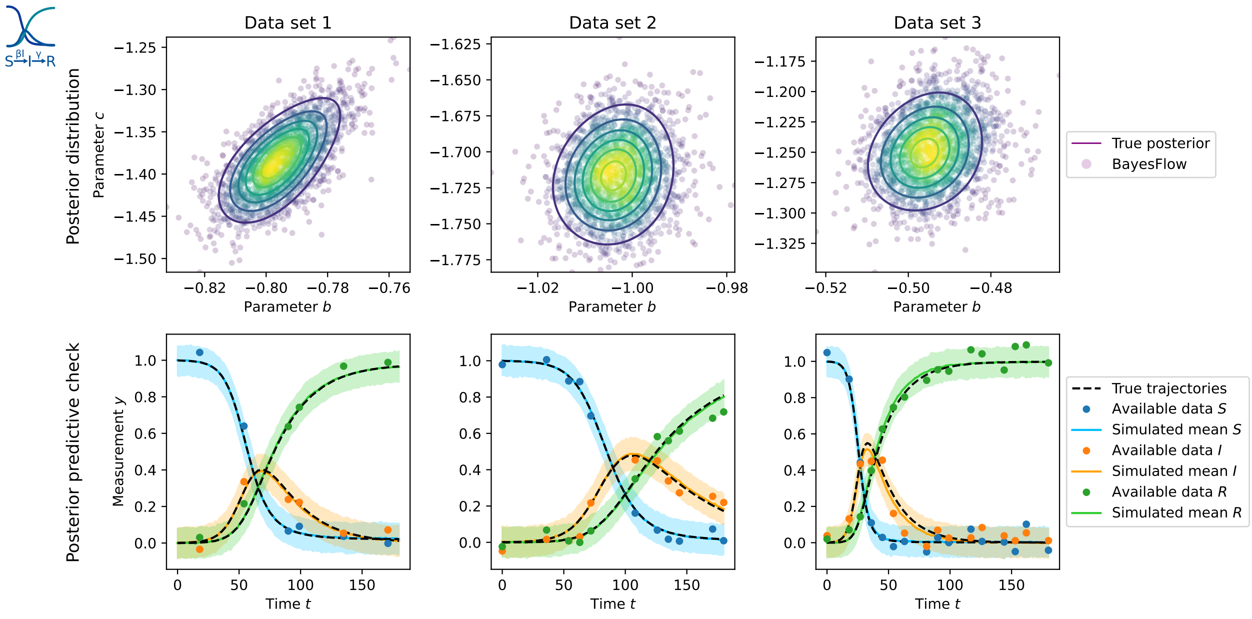

(a) Posterior samples and predictive checks using “Insert −1”

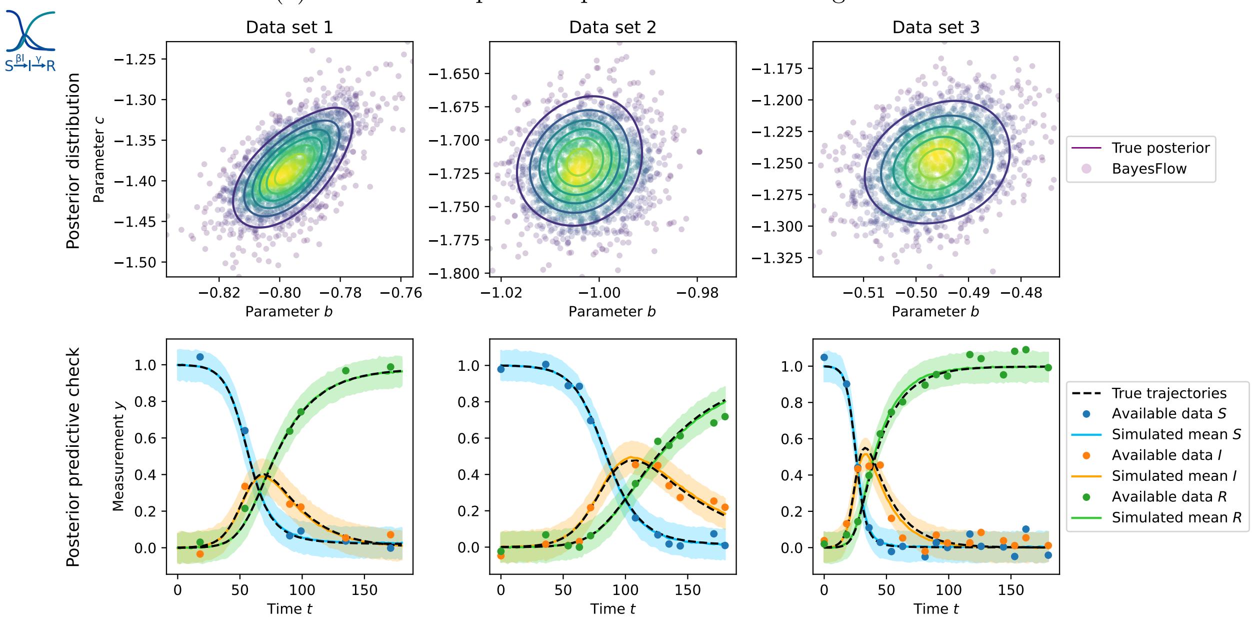

(b) Posterior samples and predictive checks using “Time labels”

Figure AA: *Results for the SIR ODE model using the encodings (a) “Insert −1”, (b) “Time labels”.* Respective top: Posterior distributions. Respective bottom: Posterior predictive checks showing the means of noise-corrupted simulations and their centered 90% credible intervals. Three data sets at ground truth parameters $[-0.8, -1.4]$ (Data set 1, left, $n_\varnothing = 15$), $[-1.0, -1.7]$ (Data set 2, middle, $n_\varnothing = 10$) and $[-0.5, -1.3]$ (Data set 3, right, $n_\varnothing = 5$) are shown.

5.6 SIR SSA model

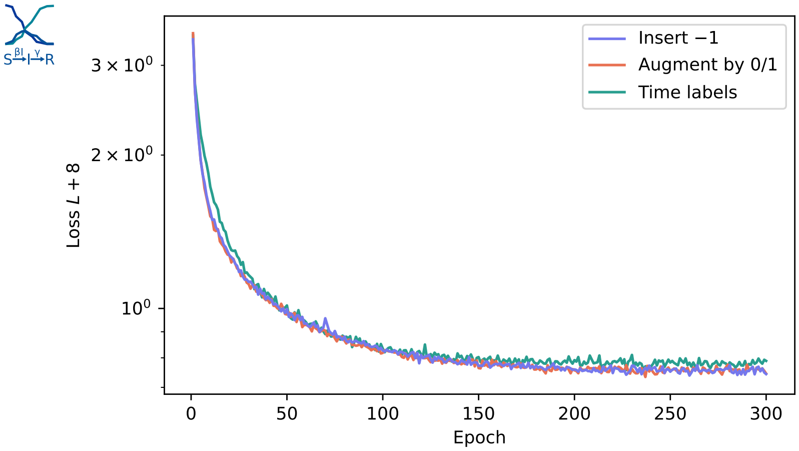

Figure AB: *Convergence plot for the SIR SSA model.* The losses of the networks trained with the encodings "Augment by 0/1" and "Insert −1" behave similarly. They converge towards a slightly lower final value than the loss for "Time labels". Due to the rather large value of $t_{\text{end}} = 50$, we used normalized time points as positional encoding for the "Time labels" approach.

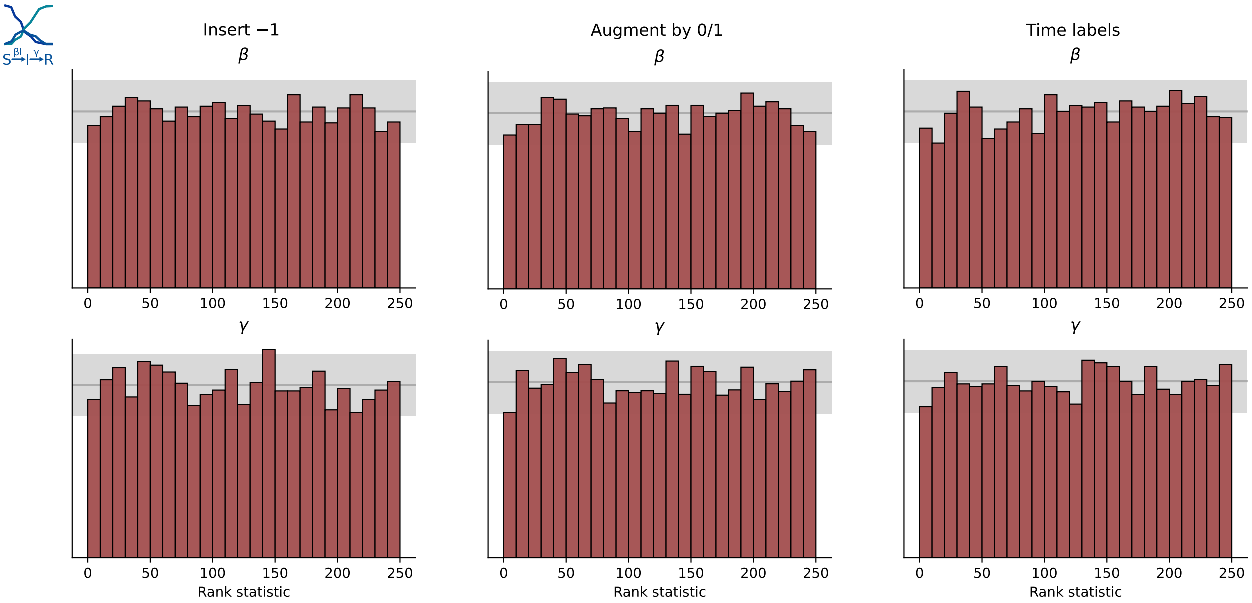

Figure AC: *SBC for the SIR SSA model.* No clear systematic bias or over-/underfitting in the posteriors is detected by the histograms.

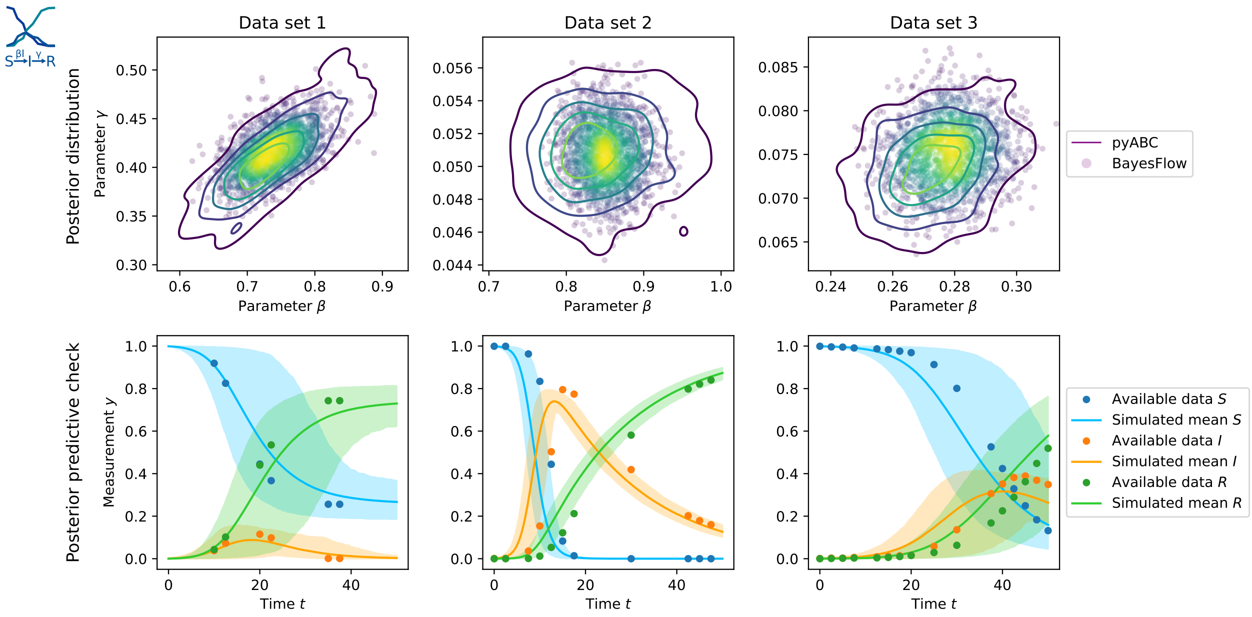

(a) Posterior samples and predictive checks using "Insert −1"

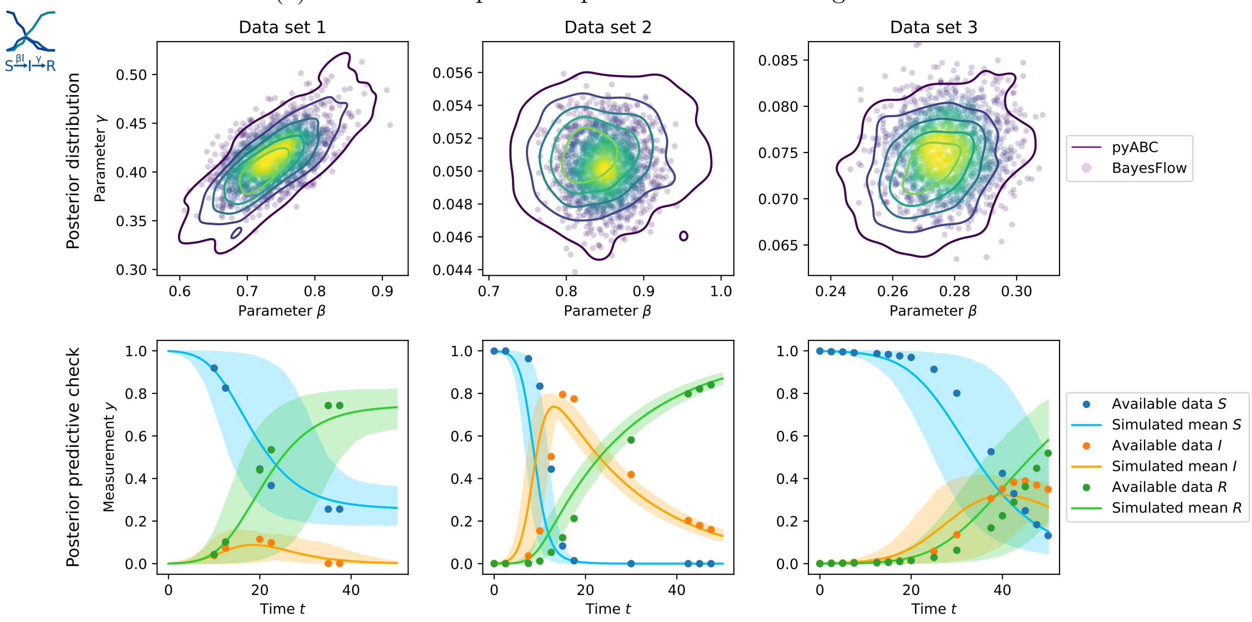

(b) Posterior samples and predictive checks using "Augment by 0/1"

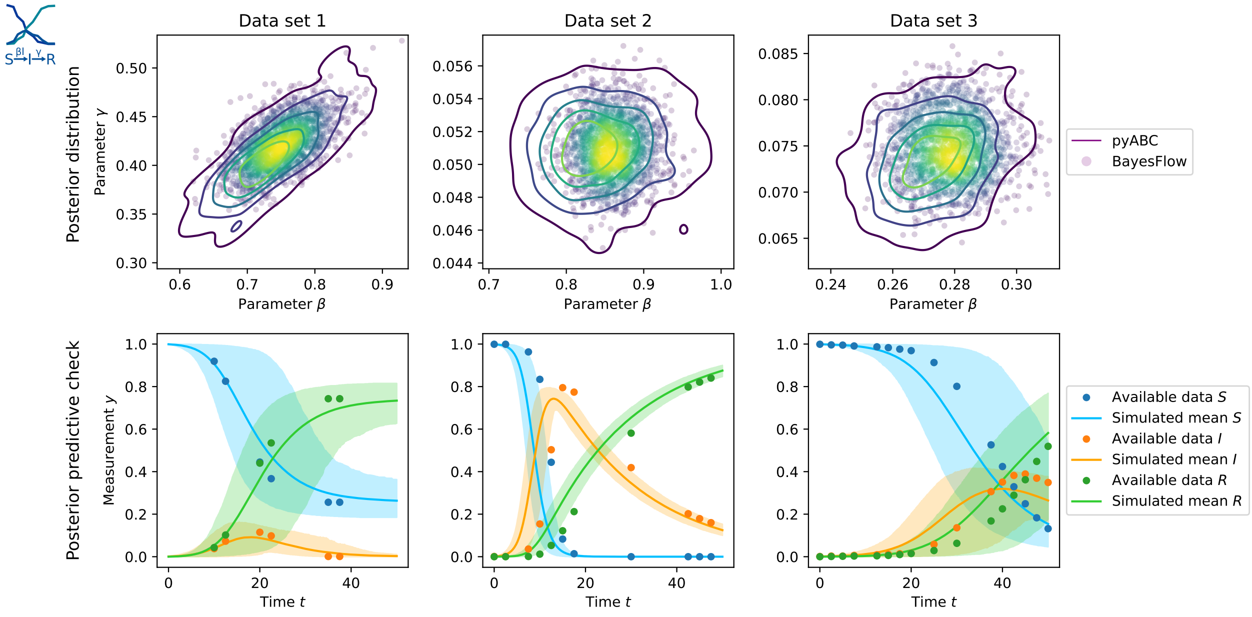

(c) Posterior samples and predictive checks using "Time labels"

Figure AD: *Results for the SIR SSA model using the encodings (a) "Insert -1", (b) "Augment by 0/1", (c) "Time labels".* Respective top: Posterior distributions. Respective bottom: Posterior predictive checks showing the means of SSA simulations and their centered 90% credible intervals. Three data sets at ground truth parameters $[0.72, 0.41]$ (Data set 1, left, $n_{\varnothing} = 15$), $[0.87, 0.05]$ (Data set 2, middle, $n_{\varnothing} = 10$) and $[0.26, 0.07]$ (Data set 3, right, $n_{\varnothing} = 5$) are shown. The three encodings yield similarly good posterior approximations, which are slightly more contracted than ones obtained from a non-exhaustive approximate Bayesian computation (ABC) run, using the tool pyABC [12]. The broad credible intervals in the predictive checks may be misleading at first glance. However, comparing with resimulations using only the ground truth parameters reveals that the data fitting achieved with our methods is already very reasonable (see Fig AE).

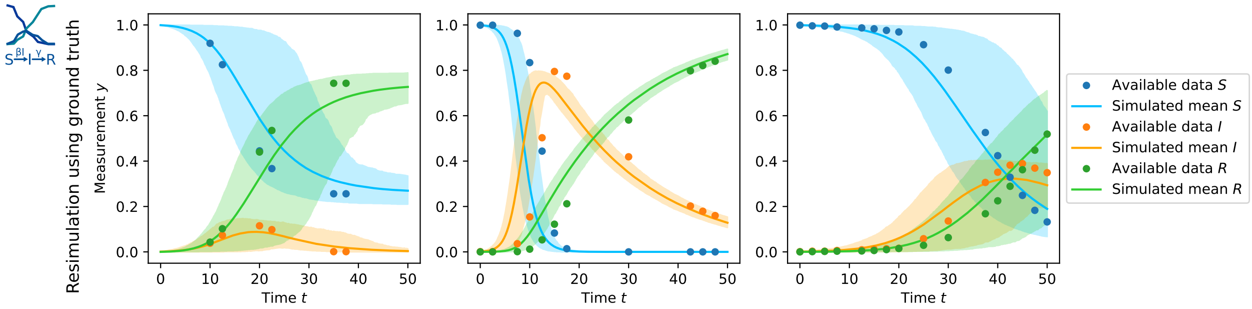

Figure AE: *Resimulations using only the ground truth for the SIR SSA model.* We learn that the broad credible intervals in the posterior predictive checks in the previous figure do not result from misapproximated posteriors, but rather from the stochasticity in our data generating process.

5.7 Conversion reaction model with parameter-dependent missingness

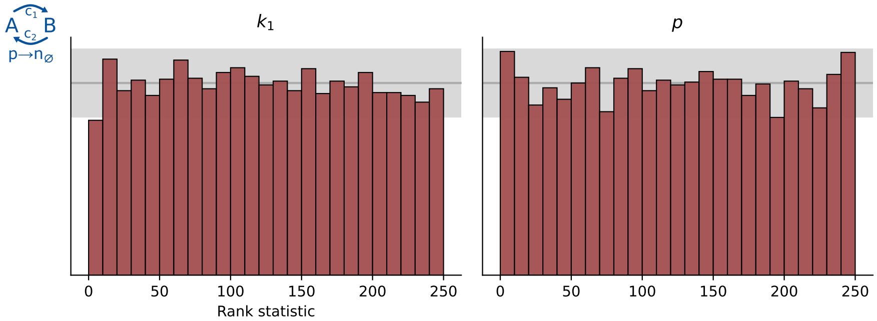

Figure AF: *SBC for the conversion reaction model with parameter-dependent missingness, using the encoding "Augment by* 0/1*".* No clear systematic bias or over-/underfitting in the posteriors is detected by the histograms.

References

[1] James Carpenter, Stuart Pocock, and Carl Johan Lamm. “Coping with missing data in clinical trials: A model-based approach applied to asthma trials”. In: *Statistics in Medicine* 21.8 (2002), pp. 1043–1066. DOI: https://doi.org/10.1002/sim.1065.

[2] Lars F. Olsen et al. “A Model of the Oscillatory Metabolism of Activated Neutrophils”. In: *Biophysical Journal* 84.1 (2003), pp. 69–81. ISSN: 0006-3495. DOI: https://doi.org/10.1016/S0006-3495(03)74833-4. URL: https://www.sciencedirect.com/science/article/pii/S0006349503748334.

[3] Nicholas T. Ingolia and Andrew W. Murray. “The Ups and Downs of Modeling the Cell Cycle”. In: *Current Biology* 14.18 (2004), R771–R777. ISSN: 0960-9822. DOI: https://doi.org/10.1016/j.cub.2004.09.018. URL: https://www.sciencedirect.com/science/article/pii/S0960982204006943.

[4] Jake Alan Pitt and Julio R. Banga. “Parameter estimation in models of biological oscillators: an automated regularised estimation approach”. In: *BMC Bioinformatics* 20.1 (2019), p. 82. ISSN: 1471-2105. DOI: 10.1186/s12859-019-2630-y. URL: https://doi.org/10.1186/s12859-019-2630-y.

[5] A. L. Hodgkin and A. F. Huxley. “A quantitative description of membrane current and its application to conduction and excitation in nerve”. In: *J. Physiol.* 117.4 (Aug. 1952), pp. 500–544.

[6] Andrea L. Bertozzi et al. “The challenges of modeling and forecasting the spread of COVID-19”. en. In: *PNAS* 117.29 (July 2020), pp. 16732–16738. ISSN: 0027-8424, 1091-6490. DOI: 10.1073/pnas.2006520117. (Visited on 10/26/2020).

[7] Elba Raimúndez et al. “COVID-19 outbreak in Wuhan demonstrates the limitations of publicly available case numbers for epidemiological modeling”. In: *Epidemics* 34 (Mar. 2021), p. 100439. ISSN: 1755-4365. DOI: https://doi.org/10.1016/j.epidem.2021.100439. URL: https://www.sciencedirect.com/science/article/pii/S1755436521000037.

[8] Ellen Kuhl. “The classical SIR model”. In: *Computational Epidemiology: Data-Driven Modeling of COVID-19*. Cham: Springer International Publishing, 2021, pp. 41–59. ISBN: 978-3-030-82890-5. DOI: 10.1007/978-3-030-82890-5_3. URL: https://doi.org/10.1007/978-3-030-82890-5_3.

[9] Stefan T Radev et al. “BayesFlow: Learning complex stochastic models with invertible neural networks”. In: *IEEE transactions on neural networks and learning systems* (2020).

[10] Daniel Foreman-Mackey et al. “emcee: The MCMC Hammer”. In: *arXiv* 125.925 (Mar. 2013), p. 306. DOI: 10.1086/670067. arXiv: 1202.3665 [astro-ph.IM].

[11] Sean Talts et al. *Validating Bayesian Inference Algorithms with Simulation-Based Calibration.* 2018. DOI: 10.48550/ARXIV.1804.06788. URL: https://arxiv.org/abs/1804.06788.

[12] Yannik Schälte et al. “pyABC: Efficient and robust easy-to-use approximate Bayesian computation”. In: *arXiv preprint arXiv:2203.13043* (2022).
