# Supplementary material for: Missing data in amortized simulation-based neural posterior estimation
Source: PLoS Comput Biol. 2024 Jun 17;20(6):e1012184. doi: 10.1371/journal.pcbi.1012184 (PMC11213359; doi:10.1371/journal.pcbi.1012184)
Supplement: Attachment — Submitted filename: Missing_Data_Amortized_Inference_ResponseLetter.pdf [file pcbi.1012184.s002.pdf]

Authors' response to editor's and reviewer's comments

For the paper: "*Missing data in amortized simulation-based neural posterior estimation*".

We thank the editor and the reviewer for their comments. Based on their remarks, we have revised the manuscript and the Supplementary Information. We addressed all their comments and resolved the corresponding issues. Our point-by-point response is provided below.

The teal boxes contain the editor's and the reviewer's comments, while the yellow boxes contain the changes made in the manuscript. To provide some context, changes (teal) are sometimes accompanied by unchanged text (black).

Response to Reviewer 1

Summary

This paper extends neural posterior estimation (NPE), in particular the BayesFlow method, to incorporate missing data. It compares 3 natural methods for doing this and, through carefully designed experiments, shows the advantage of one particular approach: augmenting the observations with binary variables indicating missingness.
My opinion is that the paper is a good contribution, acting as a proof of concept for this methodology using synthetic examples. I'd like to see a comparison with existing methods, such as multiple imputation, on real data. But I'm happy for this to be done as future work, as the authors describe in the conclusion.
Comments and suggested changes are listed below. I think these should all be easy to address.

We thank the reviewer for the feedback and the positive assessment of the quality.

As suggested, we made some modifications to the manuscript and the Supplementary Information, as detailed in the point-to-point responses below.

Major comments

(1) Algorithm 1 uses an online learning approach. Every training step generates a batch of data, which is then discarded. The authors should justify why they prefer this to an offline approach of generating the training data first and reusing it across multiple training epochs, which is discussed as an option in the Bayesflow paper and seems more efficient to me.

We thank the reviewer for pointing out the need for justification. We have elaborated on the choice of training approach in Section 2.3 as follows:

Changes in the manuscript:

Initial submission:
The entire algorithm for training and inference using BayesFlow with missing data is presented in Algorithm 1.

Revised submission:
The entire algorithm for training and inference using BayesFlow with missing data is presented in Algorithm 1. We use an online learning approach similarly to Radev et al. (2020), meaning that a batch of new data is simulated in each iteration. This is reasonable, since model simulations in our test problems are relatively fast, and effective to prevent overfitting. If simulations are more expensive, one should consider approaches where simulated data are reused to speed up training, possibly combined with regularization techniques.

Further, we extended the Discussion as follows:

Changes in the manuscript:

Initial submission:
Instead of sampling the missingness pattern individually per model simulation, one could generate multiple data sets with missing entries from a single full simulation. Especially for expensive mechanistic models, this could be useful.

Revised submission:
Instead of sampling the missingness pattern individually per model simulation, one could generate multiple data sets with missing entries from a single full simulation. Especially for expensive mechanistic models, this could be useful. Moreover, this could be easily combined with an offline training approach, where, in contrast to the online training approach employed throughout this study, simulated data using the mechanistic model are generated only once before the analysis. In such an offline approach, missing entries for each full simulation (and more generally parts of the model simulation that are computationally inexpensive) could be generated anew in each generation to avoid overfitting while keeping the computational advantages of offline training.

(2) Line 63: "Our presented approach is agnostic of the underlying missingness mechanism". This is not correct, as you later assume missingness indicators are sampled from $\pi(\tau)$, which is the definition of MCAR. The methodology could be extended to MAR and MNAR by using $\pi(\tau|x)$, but this is not done in the paper. Also application to MNAR would be challenging (as usual in missing data problems) because it would require correctly specifying the distribution of missingness indicators given unobserved data values.

We thank the reviewer for this comment. Indeed, the statement referred to is too broad and does further, especially in this brevity, not belong in the "related work" section. Thus, we have removed it from there:

Changes in the manuscript:

Initial submission:
Our presented approach is agnostic of the underlying missingness mechanism, as long as the network learned to recognize what data are missing or present.

Revised submission:
-

To clarify the important point of the assumptions we make on the missingness distribution, we have extended the Discussion as follows:

Changes in the manuscript:

Initial submission:

-

Revised submission:

Last, in this study we have considered entries missing completely at random (MCAR), or with the portion of missing entries dependent on latent parameters. Inspection of the derivation of our approach in Section 2.3 however reveals that no assumptions on the missingness distribution $\pi(\tau)$ need to be made, as it is completely simulation-based. In particular, this conceptually allows the consideration of general data-dependent missingness patterns $\pi(\tau|x)$ (MNAR). However, the practical feasibility of this remains to be tested. As common in missing data problems, an application to MNAR can be challenging, as it requires an accurate specification of the distribution of missing values given observable data.

(3) I liked the observation in Section 3.2 that your approach can improve on BayesFlow even outside the context of missing data as it's usually conceived. That is, it improves convergence for data of variable length. You could consider highlighting this as a secondary contribution of the paper in the abstract/introduction.

We thank the reviewer for this comment. We do believe it is a valuable insight that, compared to original BayesFlow, our approach shows improved convergence in the case of time series data of variable length by treating them as a special case of missing data. This insight is now highlighted in the abstract and the introduction.

Changes in the abstract:

We find that an approach in which the data vector is augmented with binary indicators of presence or absence of values performs the most robustly.

Revised submission:

We find that an approach in which the data vector is augmented with binary indicators of presence or absence of values performs the most robustly. Indeed, it improved the performance also for the simpler problem of data sets with variable length.

Changes in the manuscript:

Initial submission:

We find that an approach in which the data matrix is augmented with binary indicators of presence or absence of values performs the most robustly. Further, we demonstrate how this approach performs advantageously also in the simpler case of time series of different length.

Revised submission:

We find that an approach in which the data matrix is augmented with binary indicators of presence or absence of values performs the most robustly. Further, we demonstrate how this approach performs advantageously also in the simpler case of time series of different length, thereby showing how BayesFlow performance might be even improved in the absence of missing data.

(4) NPE methods can be inaccurate under misspecified models (see for instance https://arxiv.org/abs/2210.06564). Adding an extra model for missingness seems to increase the potential for misspecification, especially in situations where MNAR is possible. I think addressing this issue is beyond the scope of this paper, but it would be good to add some discussion of it.

We thank the reviewer for this comment. We have added a discussion of this just behind our response to comment (2):

Changes in the manuscript:

Initial submission:
-

Revised submission:
In general for neural posterior estimation, adequacy of the learned posterior distribution across the domain of possible observed data and parameters, as well as adequacy of the employed model to describe the observed data must be carefully assessed. For this, there exist established systematic approaches, whose application in the context of missing data would however remain to be checked (Radev et al. 2020; Schmitt et al. 2023).

Minor comments

(5) Line 43: "Possible reasons exist plenty" - is this a typo?

We thank the reviewer for noticing this typo. We have corrected this typo:

Changes in the manuscript:

Initial submission:
Possible reasons exist plenty, including incomplete entry, data loss, device malfunction, or study participant non-response.

Revised submission:
There are many possible reasons, including incomplete entry, data loss, device malfunction, or study participant non-response.

(6) Line 76: "imputation... holds no conceptual advantage over approaches discarding missing data". I don't think this is correct in general. For instance see the discussion on the limitations of deletion methods in Chapter 1 of "Flexible Imputation of Missing Data" by van Buuren. I suspect the authors may be referring to the case of likelihood-based inference under ignorable missingness.

We thank the reviewer for this comment, as it reveals that our wording was inaccurate. What we meant to say is that imputation cannot create information, and thus imputing values holds no advantage over not imputing missing values, i.e. discarding them, as long as together with the discarded missing data no further data are discarded (e.g. incomplete rows). As our approach simply masks missing data and removes no further data, it thus does not lose information. In contrast, approaches such as listwise deletion clearly lose information.

In general, the proper study of the effect of imputation would be very interesting. Depending on the imputation approach, the measured data and prior knowledge are used for imputing. This means that imputed values depend on the measured data and some prior. This dependence needs to be properly considered in the inference process to avoid over-confidence in the estimates. We hypothesize and provide theoretical arguments (see Supplementary Information, Section 3) that properly accounting for this would limit the information gain, but a computational analysis remains to be done.

We have clarified this point as follows:

Changes in the manuscript:

Initial submission:
Strategies to deal with missing data can be broadly divided into methods discarding entries with missing values, and approaches replacing missing values with imputed values. Imputation strategies include e.g. mean, maximum-likelihood, and multiple imputation, and deep learning approaches using e.g. LSTM networks (Song et al. 2020) and autoencoders (Khadka and Shakya 2020), see Nazabal et al. (2020) and Joel, Doorsamy, and Paul (2022) for a review. In the context of neural posterior estimation, Lueckmann et al. (2017) developed an integrated approach to simultaneously learn a model predicting parameters with failing simulations, and impute missing data. However, the faithful reconstruction of missing data using (multiple or weighted) imputation approaches relies on the accuracy of the used imputation method (see also a discussion of the inadequacy of overly simple, e.g. linear, imputation methods in the Supplementary Information, Section 2). Moreover, when properly accounting for uncertainty, imputation does not change the effective parameter posterior distribution, and therefore holds no conceptual advantage over approaches discarding missing data (see the Supplementary Information, Section 3). Instead, our approach falls into the first above category, i.e. it discards missing values.

Revised submission:
Strategies to deal with missing data can be broadly divided into methods discarding entries with missing values, and approaches replacing missing values with imputed values. Imputation strategies include e.g. mean, maximum-likelihood, and multiple imputation, and deep learning approaches using e.g. LSTM networks (Song et al. 2020) and autoencoders (Khadka and Shakya 2020), see Nazabal et al. (2020) and Joel, Doorsamy, and Paul (2022) for a review. In the context of neural posterior estimation, Lueckmann et al. (2017) developed an integrated approach to simultaneously learn a model predicting parameters with failing simulations, and impute missing data. However, the faithful reconstruction of missing data using (multiple or weighted) imputation approaches relies on the accuracy of the used imputation method (see also a discussion of the inadequacy of overly simple, e.g. linear, imputation methods in the Supplementary Information, Section 2). Moreover, when the uncertainty of imputed values is properly accounted for, by considering their distribution that depends on the available values and prior knowledge leveraged for imputation, parameter estimation from imputed data should yield the same effective posterior distribution as parameter estimation considering only the available data, provided the underlying missingness mechanism is captured accurately in the model (see the Supplementary Information, Section 3). Therefore, in such a Bayesian sense, there should be no conceptual advantage of using imputation over approaches discarding missing data, as long as they do not discard further data. Unfortunately, as many established inference methods cannot deal with missing (not-available, NA) entries, methods discarding missing entries often discard entire cases, such as listwise deletion (Pepinsky 2018), in which case they lose information. Our approach also discards missing values; however, importantly, it does not discard any non-missing data, and therefore neither loses information nor introduces a potential bias due to inadequacy of an imputation method.

(7) Line 121: I think the equation involving NA implicity assumes that 0*NA=0. It would be worth pointing this out. Often the convention is that 0*NA results in NA. For instance the R language does this.

We thank the reviewer for this comment. We have clarified what we mean by multiplying NA with a binary indicator. Note that we have discarded the explanation "$\odot$ denotes the Hadamard product" as this now occurs earlier in the paper due to changes made in response to (9):

Changes in the manuscript:

Initial submission:
In the case of incomplete data, the vector x^{obs} is not available completely, but some of its entries are missing, giving observed data $x^{\text{obs}}_{\text{avai}} \in (\mathbb{R} \cup \{\text{NA}\})^{n_x}$. Explicitly, defining the binary availability mask $\tau \in \{0, 1\}^{n_x}$, where 0 indicates absence and 1 presence of a data point, we consider $x^{\text{obs}}_{\text{avai}} = x^{\text{obs}} \odot \tau^{\text{obs}} + \text{NA} \cdot (1 - \tau^{\text{obs}})$, where $\odot$ denotes the Hadamard product.

Revised submission:
In the case of incomplete data, the vector x^{obs} is not available completely, but some of its entries are missing, giving observed data $x^{\text{obs}}_{\text{avai}} \in (\mathbb{R} \cup \{\text{NA}\})^{n_x}$. Explicitly, defining the binary availability mask $\tau^{\text{obs}} \in \{0, 1\}^{n_x}$, where 0 indicates absence and 1 presence of a data point, we consider $x^{\text{obs}}_{\text{avai}} = x^{\text{obs}} \odot \tau^{\text{obs}} + \text{NA} \cdot (1 - \tau^{\text{obs}})$, where the convention $\text{NA} \cdot 1 = \text{NA}$ and $\text{NA} \cdot 0 = 0$ is used.

(8) Line 128: "Simply dropping them from the data vector is no solution." This sounds like the basis of deletion methods, which are a solution to the problem and sometimes work well. Perhaps the authors mean that this solution wouldn't work **in the context of amortized inference**, as they argue in Section 1 of the supplement.

We thank the reviewer for this comment. We have specified the statement accordingly:

Changes in the manuscript:

Initial submission:
Missing values ("not available", "not a number") cannot be handled by neural networks, as they result in failing cost function and gradient evaluations. Simply dropping them from the data vector is no solution, as the context of which data were present would be lost.

Revised submission:
Missing values ("not available", "not a number") cannot be handled by neural networks, as they result in failing cost function and gradient evaluations. Simply dropping them from the data vector is no solution in the context of amortized inference, as the information about which data were present would be lost.

(9) Section 3: I couldn't find a description of what type of cINN is used, only the number of layers.

We thank the reviewer for pointing out the lack of clarity. We have added the following section to explain the architecture of the cINN employed:

Changes in the manuscript:

Initial submission:

-

Revised submission:

2.1.2 Invertible architecture

The basic unit of the cINN in BayesFlow is the affine coupling layer (Dinh, Sohl-Dickstein, and Bengio 2017; Radev et al. 2020). A layer comprises four internal functions s_1, t_1, s_2, t_2, each realized as fully connected neural networks with exponential linear units. In the forward direction, the input vector u is split into two halves u_1 and u_2, which then undergo a sequence of operations to give the output $v = (v_1, v_2)$:

$$v_1 = u_1 \odot \exp(s_1(u_2)) + t_1(u_2), \quad v_2 = u_2 \odot \exp(s_2(v_1)) + t_2(v_1)$$

Here, $\odot$ denotes the Hadamard product and $\exp(\cdot)$ the elementwise exponential function. The non-linear mapping $u \mapsto v$ is bijective, with the inverse given by:

$$u_2 = (v_2 - t_2(v_1)) \odot \exp(-s_2(v_1)), \quad u_1 = (v_1 - t_1(u_2)) \odot \exp(-s_1(u_2))$$

In practice, multiple layers are stacked to craft an invertible chain with sufficient expressiveness. The input to the first layer are the parameters θ of interest, and the output of the final layer are the latent variables z. To condition the mapping on the data x, either x or summary statistics $h = h_\psi(x)$ thereof are additionally fed as input into all internal networks. The chain of conditional layers yields the cINN f_ϕ, which is trained to learn the normalizing flow from θ to z using information from the data. The complete forward and inverse pass through the cINN can be written as $z = f_\phi(\theta; h)$ and $\theta = f_\phi^{-1}(z; h)$, respectively.

(10) Line 192: You say the true posterior is the distribution given only the observed data. It's worth noting that the reason this is true is that you assume ignorable missingness.

We thank the reviewer for this comment. We have added the following clarification:

Changes in the manuscript:

Initial submission:
When comparing results to the "true posterior", we imply the distribution $\pi(\theta|x^{\text{obs}}_{\text{avai}})$ given only actually observed data, ignoring missing entries.

Revised submission:
When comparing results to the "true posterior", we imply the distribution $\pi(\theta|x^{\text{obs}}_{\text{avai}})$ given only actually observed data, ignoring missing entries, as we assume ignorable missingness in the considered problems.

(11) Figure 3: The bottom row might be more easily interpretable if you used the same y-axis in all plots.

We thank the reviewer for this suggestion. We agree that using the same y-axis would make the bottom row of the figure more easy to interpret. We have updated the figure accordingly:

Changes in the manuscript:

Initial submission:

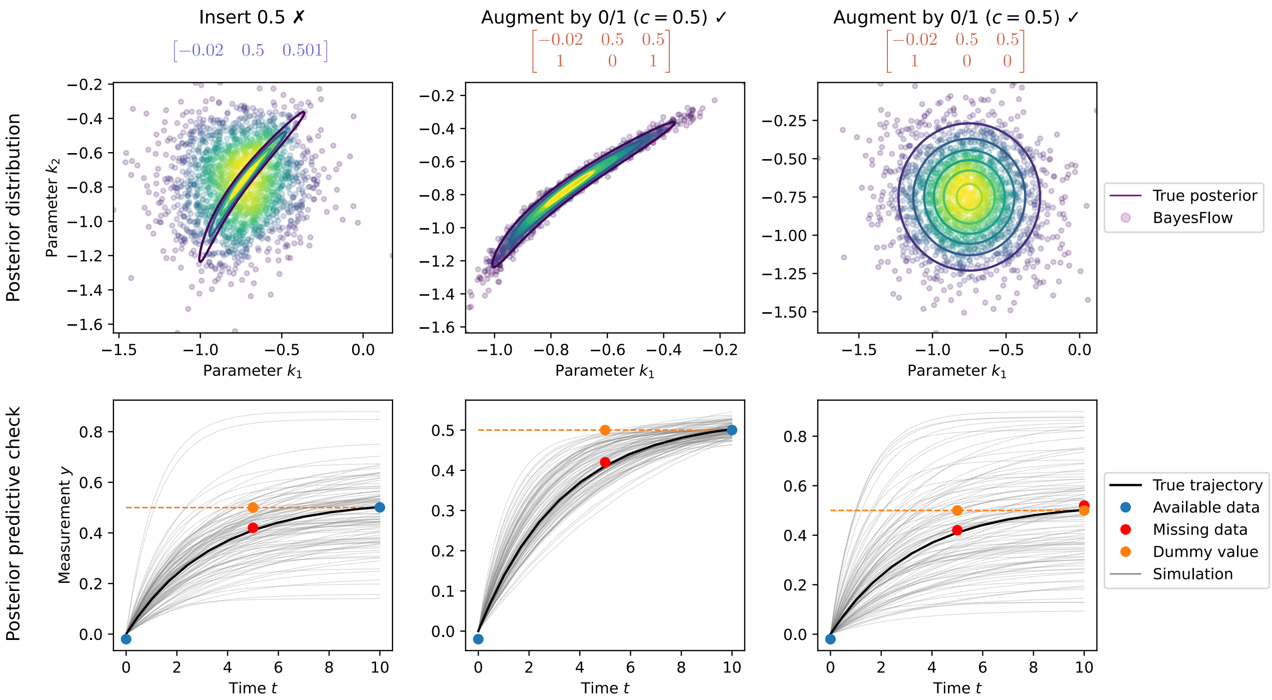

Revised submission:

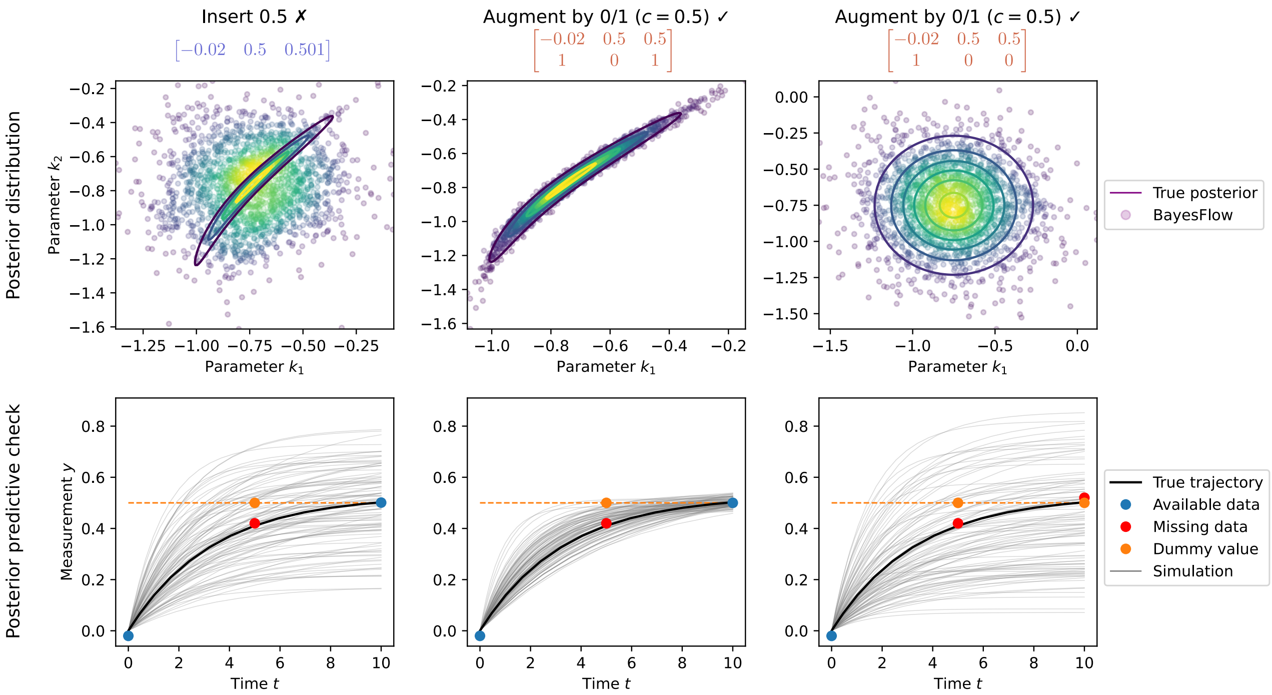

(12) Line 247: "This gives a mathematically inexact approximation of the loss function, as its true value is only approximated as an average over iterations, but the network parameters are updated after each iteration." I didn't fully understand this sentence. In particular stochastic gradient methods always use inexact approximations of the loss. I think the key point here is that fixing the number of observations within a batch means the loss estimate (1) remains unbiased but (2) has higher variance (see `https://en.wikipedia.org/wiki/Law_of_total_variance`).

We thank the reviewer for this comment. We agree that the sugguested formulation is a

more precise description of the difference between fixing the number of missing values within a batch and sampling this number for each individual data set. We have adapted the statement accordingly:

Changes in the manuscript:

Initial submission:
This can be explained by the fact that in the original BayesFlow method the number of available data points is sampled only once per batch, to enable vectorized operations. This gives a mathematically inexact approximation of the loss function, as its true value is only approximated as an average over iterations, but the network parameters are updated after each iteration. This leads to a loss that fluctuates severely from one iteration to another (Figure 4b), which may then affect the convergence of the stochastic gradient descent algorithm (Figure 4a). Contrarily, the approach "Augment by 0/1" yields augmented data sets of fixed size, thus it is possible to sample the number of available data points, or here rather the time series length, on the level of individual data sets and thereby target the same distribution in each iteration.

Revised submission:
This can be explained by the fact that in the original BayesFlow method the number of available data points is sampled only once per batch, to enable vectorized operations. This results in a loss function estimate with substantially higher variance (although it remains unbiased). Its severe fluctuation over iterations (Figure 4b) may then affect the convergence of the stochastic gradient descent algorithm (Figure 4a). Contrarily, the approach "Augment by 0/1" yields augmented data sets of fixed size, thus it is possible to sample the number of available data points, or here rather the time series length, on the level of individual data sets and thereby calculate loss estimates with reduced variance.

Further, we have updated a related statement in Section 2.3 as follows:

Changes in the manuscript:

Initial submission:
Sampling the number of missing data points on individual instead of batch level can be hoped to improve the stability of gradient descent when training the network parameters. This is because when using batch-level missingness sampling, the expected cost function varies over batches, that is we effectively use a different cost function in each step.

Revised submission:
Sampling the number of missing data points on individual instead of batch level reduces the variance of the cost function approximation across batches (i.e. iterations), which can be hoped to improve the stability of gradient descent when training the network parameters.

(13) Supplement line 63: I think the last $\pi(x^{\text{obs}})$ on this line should have a subscript "avai".

We thank the reviewer for noticing this mistake. We have added the subscript "avai" to the expression:

Changes in the Supplementary Information:

Initial submission:
In this, either the imputation method is biased (if available, towards additional information), or it should be a faithful approximation $p(x^{\text{obs}}|x^{\text{obs}}_{\text{avai}}) \approx \pi(x^{\text{obs}}|x^{\text{obs}}_{\text{avai}})$, where $\pi(x^{\text{obs}}|x^{\text{obs}}_{\text{avai}})\pi(x^{\text{obs}}) = \pi(x^{\text{obs}}_{\text{avai}}, x^{\text{obs}})$ is the joint distribution of missing and complete data.

Revised submission:
In this, either the imputation method is biased (if available, towards additional information), or it should be a faithful approximation $p(x^{\text{obs}}|x^{\text{obs}}_{\text{avai}}) \approx \pi(x^{\text{obs}}|x^{\text{obs}}_{\text{avai}})$, where $\pi(x^{\text{obs}}|x^{\text{obs}}_{\text{avai}})\pi(x^{\text{obs}}_{\text{avai}}) = \pi(x^{\text{obs}}_{\text{avai}}, x^{\text{obs}})$ is the joint distribution of missing and complete data.

(14) Supplement, just after line 67: "the missingness pattern contains information about the parameters beyond the complete data". I'm not a missing data expert, but this seems like an unusual case. It's typically to assume that $\pi(\tau|x, \psi)$ i.e. missingness pattern can depend on the full data and parameters ψ which are separate from the model parameters θ (in Bayesian setting typically ψ, θ are assumed to be a priori independent). Assuming also a direct dependence of τ on θ is mathematically possible, but I think the authors should make a case for why this would be a useful model.

We thank the reviewer for this comment. A case in which the missingness is informative about the model parameters are clinical trials. Individuals participating in clinical trials might decide to discontinue the participation. This is more likely if they have side effects or do not respond favorably to a drug.

We have added the example to the Supplement to provide additional context:

Changes in the Supplementary Information:

Initial submission:
If instead $\pi(\theta|x^{\text{obs}}) \neq \pi(\theta|x^{\text{obs}}, x^{\text{obs}}_{\text{avai}})$, i.e. the missingness pattern contains information about the parameters beyond the complete data, we would still need to pass the missingness pattern to the amortized posterior sampling method, complicating its training. In doing so, we would however similarly finally obtain a marginalized posterior approximation

$$\int \pi(\theta|x^{\text{obs}}, x^{\text{obs}}_{\text{avai}})p(x^{\text{obs}}|x^{\text{obs}}_{\text{avai}})\, dx^{\text{obs}} = \pi(\theta|x^{\text{obs}}_{\text{avai}}),$$

i.e. recover the posterior given the available data (including their missingness pattern) again.

Revised submission:
If instead $\pi(\theta|x^{\text{obs}}) \neq \pi(\theta|x^{\text{obs}}, x^{\text{obs}}_{\text{avai}})$, i.e. the missingness pattern contains information about the parameters beyond the complete data, we would still need to pass the missingness pattern to the amortized posterior sampling method, complicating its training. In doing so, we would however similarly finally obtain a marginalized posterior approximation

$$\int \pi(\theta|x^{\text{obs}}, x^{\text{obs}}_{\text{avai}})p(x^{\text{obs}}|x^{\text{obs}}_{\text{avai}})\, dx^{\text{obs}} = \pi(\theta|x^{\text{obs}}_{\text{avai}}),$$

i.e. recover the posterior given the available data (including their missingness pattern) again. An application example in which missingness is known to be informative are clinical trials (Carpenter, Pocock, and Johan Lamm 2002). Participants might decide to drop out before a trial is concluded. The likelihood of this missingness occurring can depend on both data (e.g. when a participant gets a negative test result and subsequently loses interest in participating in follow-up tests) and model parameters (e.g. when a participant has a precondition that renders them more susceptible to side effects). Accordingly, in the latter case, the missingness pattern carries information about the parameters.

References

Carpenter, James, Stuart Pocock, and Carl Johan Lamm (2002). "Coping with missing data in clinical trials: A model-based approach applied to asthma trials". In: Statistics in Medicine 21.8, pp. 1043–1066. DOI: https://doi.org/10.1002/sim.1065.

Dinh, Laurent, Jascha Sohl-Dickstein, and Samy Bengio (2017). "Density estimation using Real NVP". In: 5th International Conference on Learning Representations, ICLR 2017, Toulon, France, Ap OpenReview.net. URL: https://openreview.net/forum?id=HkpbnH9lx.

Joel, Luke Oluwaseye, Wesley Doorsamy, and Babu Sena Paul (2022). "A Review of Missing Data Handling Techniques for Machine Learning". In: International Journal of Innovative Technology a 5.3, pp. 971–1005.

Khadka, Shyam Krishna and Subarna Shakya (2020). "Imputing Block of Missing Data Using Deep Autoencoder". In: International Conference on Mobile Computing and Sustainable Informatics. Springer, pp. 697–707.

Lueckmann, Jan-Matthis et al. (2017). "Flexible statistical inference for mechanistic models of neural dynamics". In: Advances in neural information processing systems 30.

Nazabal, Alfredo et al. (2020). "Handling incomplete heterogeneous data using vaes". In: Pattern Recognition 107, p. 107501.

Pepinsky, Thomas B. (2018). "A Note on Listwise Deletion versus Multiple Imputation". In: Political Analysis 26.4, 480â488. DOI: 10.1017/pan.2018.18.

Radev, Stefan T et al. (2020). "BayesFlow: Learning complex stochastic models with invertible neural networks". In: IEEE transactions on neural networks and learning systems.

Schmitt, Marvin et al. (2023). "Detecting model misspecification in amortized Bayesian inference with neural networks". In: DAGM German Conference on Pattern Recognition. Springer, pp. 541–557.

Song, Wei et al. (2020). "A time series data filling method based on LSTMâtaking the stem moisture as an example". In: Sensors 20.18, p. 5045.
